# Recent Findings in the Regulation of Programmed Death Ligand 1 Expression

**DOI:** 10.3389/fimmu.2019.01337

**Published:** 2019-06-14

**Authors:** Xiangfeng Shen, Lihong Zhang, Jicheng Li, Yulin Li, Yishu Wang, Zhi-Xiang Xu

**Affiliations:** ^1^Key Laboratory of Pathobiology, Ministry of Education, Norman Bethune College of Medicine, Jilin University, Changchun, China; ^2^Department of Physiology, College of Basic Medical Sciences, Jilin University, Changchun, China

**Keywords:** PD-L1 expression, intrinsic signals, extrinsic signals, anti-PD-1/PD-L1, immune checkpoint therapy

## Abstract

With the recent approvals for the application of monoclonal antibodies that target the well-characterized immune checkpoints, immune therapy shows great potential against both solid and hematologic tumors. The use of these therapeutic monoclonal antibodies elicits inspiring clinical results with durable objective responses and improvements in overall survival. Agents targeting programmed cell death protein 1 (PD-1; also known as PDCD1) and its ligand (PD-L1) achieve a great success in immune checkpoints therapy. However, the majority of patients fail to respond to PD-1/PD-L1 axis inhibitors. Expression of PD-L1 on the membrane of tumor and immune cells has been shown to be associated with enhanced objective response rates to PD-1/PD-L1 inhibition. Thus, an improved understanding of how PD-L1 expression is regulated will enable us to better define its role as a predictive marker. In this review, we summarize recent findings in the regulation of PD-L1 expression.

## Introduction

With rapid development of immunotherapy for cancer treatment in decades, immune checkpoint therapy that mediates tumor cell death through the reactivated immune system has become the most attractive strategy for cancer therapy due to their impressive therapeutic efficacy. Immune checkpoints, a cluster of immune inhibitory receptors and their reciprocal ligands that negatively regulate the immune system function, are important for avoiding autoimmunity and for protecting collateral tissue from damage under physiological conditions. On the other hand, cancer cells could also make use of immune checkpoints to inhibit the activity of T cells, leading to the immune escape of tumors.

The role of immune checkpoints in the suppression of T cell activity has led to the development of immune checkpoint inhibitors in the treatment of cancer. Among the immune checkpoints, programmed cell death protein 1 (PD-1) and its ligand PD-L1 have stood out because of their proven value as therapeutic targets in a large number of malignancies. Inhibition of the PD-1/PD-L1 axis contributes to important clinical advances in cancer therapy, including melanoma (1, 2), non-small cell lung cancer (NSCLC) (3–5), renal cell carcinoma (RCC) (6), Hodgkin's lymphoma (7, 8), bladder cancer (9, 10), head and neck squamous cell carcinoma (HNSCC) (11–13), Merkel-cell carcinoma (14), urothelial carcinoma (15), and microsatellite instable-high (MSI-H) or mismatch repair-deficient (dMMR) solid tumors (16–18). However, despite the considerable improvement in patient outcome that has been achieved with PD-1/PD-L1 blockade, the durable objective responses to the checkpoint therapeutics are various among different tumor types and limited in only a minority of patients. Intra-tumoral PD-L1 expression is generally associated with a better response to PD-1/PD-L1 blockade in patients across multiple cancer types (3, 19–21). Therefore, in order to predict the efficiency and optimize the anti-PD-1/PD-L1 therapy alone or in combination, improving the understanding of the regulatory mechanisms of PD-L1 in cancer should be of utmost importance for not only identifying its role as biomarker but also for designing the synergistic treatment combinations. Here, we focus on the current knowledge of PD-L1 regulation.

## Anti-PD-1/PD-L1 Inhibitors as Immune Checkpoint Therapy

Although the immune system of our body should eliminate cancer cells as “foreign,” the interactions between the immune system and cancer cells are complex. A natural balance called tolerance may be reached between immune response and cancer, where cancer cells are seen as “self.” Furthermore, tumor cells may evade immune destruction by suppressing immunity through multiple mechanisms, thus called immune escape. T cell immunity, especially activation of cytotoxic T lymphocyte (CTL) determines the ultimate amplitude and quality of antitumor immune response. Signals from T cell receptor, which recognizes antigen along with the major histocompatibility complex (also known as human leukocyte antigen) presented on the surface of antigen-presenting cell (APC), and additional co-stimulatory signals provided by the engagement of CD28 on the T cell surface with B7 molecules (CD80 and CD86) on the APC, are required for T cell activation (22, 23). Activated T cells attack and eventually destroy tumor cells that express tumor-specific antigens as “foreign.”

In addition to initiating proliferation and functional differentiation, T cell activation also induces the stimulation of inhibitory pathways (also known as checkpoint pathways), which eventually attenuate and terminate T cell responses (24–26). Many types of solid tumors generate an immunosuppressive microenvironment to avoid the destiny of being lysed by CTL through the inhibitory ligand called PD-L1 that is expressed on the surface of tumor cells (27, 28). PD-1 (CD279), which belongs to the CD28 family, is encoded by the PDCD1 gene located on chromosome 2q37.3 and is mainly expressed on activated T cells (29). It has two ligands, PD-L1 (CD274, B7-H1) and PD-L2 (CD273, B7-DC), with different expression patterns, which are, respectively, encoded by the *CD274* and *PDCD1LG2* genes located on chromosome 9p24.1. PD-L1 is expressed abundantly on immune cells (e.g., T cells, B cells, dendritic cells (DCs), and macrophages) and parenchymal tissue cells (mesenchymal stem cells, epithelial, endothelial cells, and brown adipocytes), as well as tumor cells. The expression of PD-L2 is considered to be mainly restricted to activated DCs and macrophages (30–33). Studies have shown that PD-1/PD-L1 axis can be hijacked by tumors as a co-inhibitory pathway to compromise the immune response toward cancer via blocking proliferation, induction of apoptosis by CTL, and promotion of regulatory T cell differentiation, which eventually induces an immunosuppressive microenvironment in tumor (25, 26). Considering that PD-L1 overexpression is a situation that is commonly seen in tumors and usually confers a poor prognosis, the therapeutic intervention targeting this co-inhibitory axis is substantially enticing to researchers and patients (34–37). Antibodies blocking the interaction between PD-1 and PD-L1 by either targeting PD-1 (pembrolizumab, nivolumab, and cemiplimab) or PD-L1 (atezolizumab, avelumab, and durvalumab) (Table 1) both induce durable objective responses in patients with melanoma (1, 2), NSCLC (3–5) and RCC (6), and other malignancies (7–15). Although the immune checkpoint therapy targeting either PD-1 or PD-L1 has been usually recognized as the same subclass in the field of tumor immunotherapy at present, PD-1 and PD-L1 blockades may differ in the mechanism of action due to the complicated subtle interactions among the immune checkpoint system. For example, in addition to PD-1, studies have reported that co-stimulatory molecule CD80 (B7-1) can also serve as a receptor for PD-L1, and the binding affinity of CD80 to PD-L1 is comparable to its affinity for CD28 (38). More importantly, the binding of PD-L1 to CD80 functionally inhibits the proliferation of T cells and promotes the apoptosis of activated CD8^+^ T cells (38, 39). Similarly, in addition to PD-L1, PD-1 also binds to its ligand PD-L2, which is expressed on solid tumor cells and hematological malignancies (40–45) and bears an impact on the anti-PD-1 therapy (41, 42, 46). Furthermore, PD-L2 has even been characterized as a novel potential therapeutic target for cancer treatment (45). Therefore, more evidence is needed to underpin the unique characteristics of PD-1 and PD-L1 inhibitors in order to achieve a better understanding of their differences.

**Table 1:** Characteristics of current FDA-approved PD-1/PD-L1 checkpoint blockades.

**Drug**	**Brand name**	**Target**	**Company**	**Time approved**	**FDA approved indications**
Pembrolizumab	Keytruda	PD-1	Merck	09/04/2014 12/18/2015 08/05/2016 03/15/2017 05/18/2017 05/10/2017 05/23/2017 09/22/2017 06/12/2018 06/13/2018	Metastatic NSCLC Unresectable or metastatic melanoma Recurrent of metastatic HNSCC Refractory cHL Advanced or metastatic urothelial carcinoma Untreated metastatic non-squamous NSCLC (combined with pemetrexed and carboplatin) Unresectable or metastatic MSI-H or dMMR solid tumors Advanced gastric cancer Recurrent or metastatic cervical cancer Refractory PMBCL
Nivolumab	Opdivo	PD-1	Bristo-Myers Squibb	12/22/2014 03/04/2015 10/09/2015 11/23/2015 05/17/2016 11/10/2016 02/02/2017 08/01/2017 09/22/2017 12/20/2017 07/10/2018	Unresectable or metastatic melanoma Metastatic squamous NSCLC Metastatic NSCLC Untreated advanced renal cell carcinoma Relapsed cHL Recurrent or metastatic HNSCC Advanced or metastatic urothelial carcinoma MSI-H or dMMR metastatic CRC HCC previously treated with sorafenib Resectable or metastatic melanoma Advanced RCC (Combined with ipilimumab)
Cemiplimab	Libtayo	PD-1	Sanofi/Regeneron	09/28/2018	Metastatic CSCC
Atezolizumab	Tecentriq	PD-L1	Genetech/Roche	05/18/2016 10/18/2016	Advanced or metastatic urothelial carcinoma Metastatic NSCLC
Avelumab	Bavencio	PD-L1	EMD Serono	03/23/2017 05/09/2017	Metastatic MCC Advanced or metastatic urothelial carcinoma
Durvalumab	Imfinzi	PD-L1	AstraZeneca	05/01/2017 02/16/2018	Advanced or metastatic urothelial carcinoma (UK Limited) Unresectable stage III NSCLC

*FDA, US Food and Drug Administration; PD-1, programmed death protein 1; PD-L1, programmed death ligand-1; NSCLC, non-small cell lung cancer; HNSCC, head and neck squamous cell carcinomas; cHL, classical Hodgkin lymphoma; MSI-H, microsatellite instability-high; dMMR, mismatch repair-deficient; PMBCL, primary mediastinal large B-cell lymphoma; CRC, colorectal cancer; HCC, hepatocellular carcinoma; RCC, renal cell carcinoma; CSCC, cutaneous squamous cell carcinoma; MCC, Merkel cell carcinoma*.

## PD-L1 Regulation in Cancer

Expression of PD-L1 is complicated and various in different tumor types. It can be regulated by various intrinsic and extrinsic signals, such as chromosomal alterations, epigenetic modifications, aberrant oncogenic and tumor suppressor signals, inflammatory cytokines, and other factors at the genetic, transcriptional, post-transcriptional, translational, and post-translational levels.

### Genetic Basis of PD-L1 Expression in Cancer

The genetic aberrations of the chromosome 9p24.1, on which *CD274* resides, represent a key mechanism affecting PD-L1 expression. Copy number alterations (CNAs) in chromosome 9p involving *PD-L1* were recently detected in 22 cancer types (47). It revealed that gains of copy numbers in chromosome 9p occur frequently in bladder, breast, cervical, colorectal, head and neck, and ovarian carcinomas, but are a rare event in pancreatic, renal cell, and papillary thyroid carcinoma. On the other hand, *PD-L1* gene deletions were found to be more frequent than *PD-L1* gains in cancers, especially in melanoma and NSCLC (>50%). Generally, overexpression of PD-L1 frequently occurs in tumors coupled with copy number gains, especially amplification of the *PD-L1* gene. Other studies also revealed high CNAs in classical Hodgkin lymphoma (cHL) and primary mediastinal B-cell lymphoma (48, 49). A recent study showed that the CNAs of *PD-L1* are also prevalent in soft-tissue sarcomas (21.1%), with higher frequency in myxofibrosarcoma (35%) and undifferentiated pleomorphic sarcoma (34%) (50). In contrast, absence or low frequency of CNAs has been reported in lung cancer (51–53) and diffuse large B-cell lymphoma (DLBCL) (54).

In addition to the CNAs, a previous study confirmed that a somatic mutation at a naturally occurring polymorphism locus, rs4143815, in the 3′ untranslated region (3′-UTR) of *PD-L1* gene is correlated with elevated PD-L1 protein expression in gastric cancer (55, 56). Another polymorphism in the promoter region of *PD-L1* was verified to upregulate *PD-L1* mRNA and protein expression by offering a binding site for transcriptional factor SP1 in gastric cancer (57). The disruption of *PD-L1* 3′-UTR was further confirmed to invariably lead to a marked elevation of aberrant *PD-L1* transcripts. Using whole-genome sequencing, Kataoka et al. (58) identified a novel genetic mechanism termed structural variants for PD-L1 overexpression in adult T cell leukemia/lymphoma (ATL), DLBCL, and gastric adenocarcinoma. These structural variants invariably generate *PD-L1* transcripts with aberrant 3′-UTR, leading to a delayed clearance of the transcripts and elevated PD-L1 expression. Furthermore, expression of 3′-UTR-truncated *PD-L1* transcripts in EG7-OVA cells contributes to tumor immune evasion in a mouse model, which is effectively inhibited by PD-1/PD-L1 blockade. Kogure and Kataoka (59) also reported that structural variants induce PD-L1 overexpression in ATL. More recently, a study in gastric cancer has revealed that *PD-L1* rs2297136 AA+AG genotype, a new polymorphism in 3′-UTR of *PD-L1*, is also correlated with the positive protein expression of PD-L1 (60).

### Epigenetic Regulation of PD-L1 Expression in Cancer

DNA methylation and post-translational histone modifications, the most foundational epigenetic events, are central mechanisms in cancer development and progression (61, 62). Both of them contribute to immunosuppressive environment within tumors by manipulating the expression of genes associated with the process of antigen presentation, immune evasion, and T-cell exhaustion (63, 64). Hypomethylating agents and histone deacetylase inhibitors enhance the processing and presentation of tumor-associated antigens and promote the expression of additional immune-related genes, chemokines, and co-stimulatory molecules (65, 66), hence reversing the immune suppression. It suggests that the efficacy of epigenetic agents is dependent, at least in part, on adaptive immune responses. Interestingly, recent studies on the role of epigenetics in immune evasion have identified a role for epigenetic modulators in the upregulation of immune checkpoint. Yang et al. (67) reported that application of hypomethylating agents elevates PD-L1, which is responsible for the resistance of patients to the original treatment. As a corollary, a blockade of the PD-1/PD-L1 axis may be an option to help overcome the resistance. On the other hand, sufficient expression of PD-L1 within tumor microenvironment is the basis for anti-PD-1/PD-L1 therapy. Deficiency of PD-L1 could contribute to “target missing” resistance (68). Treatments leading to the upregulation of PD-L1 within a tumor may sensitize PD-1/PD-L1 checkpoint therapy. Therefore, it is possible that combined application of epigenetic therapies with PD-1/PD-L1 inhibitors will exhibit a synergy antitumor effect through the altered expression of PD-L1, as well as the host immune response.

#### DNA Methylation

Recent studies suggested that the DNA methylation status of the *PD-L1* promoter can serve as a prognostic biomarker in various malignancies (69–72). There are robust data to support that DNA methylation plays a fundamental role within the dynamic expression of the PD-L1. In melanoma, both the changes in global methylation and the DNA methylation of CpG loci in the *PD-L1* promoter are involved in regulating the expression of PD-L1 (72, 73). Consistently, hypomethylation of the *PD-L1* promoter was found to be inversely correlated with both mRNA and protein expression in HNSCC (74). In glioma, the cancer genome atlas (TCGA) data showed that cytosolic NADP^+^-dependent isocitrate dehydrogenase 1 (IDH1) mutation tumors represent an attenuated level of PD-L1 expression accompanied by higher *PD-L1* gene promoter methylation (75, 76). Supportively, the addition of 2-hydroxyglutarate, key production of IDH1 mutation, transiently showed efficacy to elevate the DNA methylation in CpG site within *PD-L1* and diminish the expression of PD-L1 (76). Moreover, it was reported that transforming growth factor β1 (TGFβ1) induces decreased expression of DNA methyltransferase 1 (DNMT1) and *PD-L1* promoter demethylation, which subsequently results in PD-L1 overexpression in lung cancer cells undergoing epithelial-mesenchymal transition (EMT) (77). These results spark significant interest in detecting the effect of hypomethylating agents on PD-L1 expression and the combination efficiency of this epigenetic therapy and anti-PD-1/PD-L1 inhibitors. As expected, decitabine, which induces the inhibition of global methylation, upregulates the transcript and protein of PD-L1 in NSCLC cell lines (78). In a study in myelodysplastic syndrome, a high level of PD-L1, PD-L2, PD-1 and cytotoxic T lymphocyte associated antigen 4 (CTLA4) expression was observed in patients treated with decitabine (67). Furthermore, the supplement of decitabine to anti-PD-1 therapy improves the efficiency of antitumor immunity (79, 80). Of note, anti-PD-1 therapy enhanced *PD-L1* promoter methylation was identified to be involved in the resistance to immune checkpoint inhibitor in NSCLC (80).

#### Histone Modifications

Histone deacetylases (HDACs) have pleiotropic efficiency in regulating immune response (81). HDAC inhibitors have been evaluated as anticancer drugs over the past two decades (82). The role of HDACs in immunotherapies has recently been investigated (65, 83–86). In melanoma, inhibition of HDAC8, a class I histone deacetylase, was proven to elevate the expression of PD-L1 via increasing the activity of a fragment of the *PD-L1* promoter (87). Another study also demonstrated the durable upregulation effect of three class I histone deacetylase inhibitors on PD-L1 due to enhanced histone acetylation of the *PD-L1* gene in melanoma (88). In contrast, Lienlaf et al. (89) have described a negative effect of HDAC6 (a class IIb histone deacetylase) inhibition or depletion on PD-L1 expression, which is mediated by activated signal transducer and activator of transcription 3 (STAT3). Both HDAC6 and STAT3 are recruited to the *PD-L1* gene promoter in melanoma. Consistently, in a study in multiple myeloma, ACY241 (an HDAC6 selective inhibitor) significantly decreases PD-L1 expression on CD138^+^ myeloma cells in patients (90). In a recent study by Booth et al. (86), two pan-HDAC inhibitors were found to decrease the expression of PD-L1 both *in vitro* and *in vivo*. The authors argued that knockdown of HDAC1 and HDAC2, members of the class I histone deacetylase family, contributes to the reduced expression of PD-L1, but not HDAC6 and HDAC10, which belong to the class IIb histone deacetylase family (86). Discrepancy of HDACs inhibitors in regulating PD-L1 expression was also reported in lung cancer (65, 83, 91).

In addition to HDACs, in a screening for epigenetic mechanisms that regulate PD-L1 expression in pancreatic cancer, histone methylation (H3K4me3) is evident to be enriched in the proximal *CD274* promoter region both *in vitro* and *in vivo*. Chromatin immunoprecipitation (ChIP) analysis revealed that MLL1, one of the H3K4 methylation-specific histone methyltransferases, is directly associated with the *CD274* promoter region. Silencing MLL1 expression dramatically decreases the H3K4me3 level in the *CD274* promoter region, leading to a decreased *PD-L1* mRNA in both human and mouse pancreatic cancer cells (92).

Whilst the regulation of DNA methylation and histone modifications in PD-L1 expression has been explored, a novel epigenetic drug, JQ1, a selective bromodomain inhibitor (BETi), was corroborated to suppress PD-L1 expression in ovarian cancer to restore cytotoxic T cell responses (93). Another study reported by Hogg et al. (94), corroborated that both constitutive and inducible expression of PD-L1 can be directly repressed at transcriptional level in different tumor cell lines and primary patient samples via decreasing the occupancy of bromodomain and extraterminal protein at the *CD274* locus. Downregulation of PD-L1 plays a crucial role in JQ1 mediated anti-cancer therapy since ectopic expression of PD-L1 blunted the therapeutic effect of JQ1. The role of BET inhibition in modulating PD-L1 expression was further confirmed by Cioffi et al. revealing that OTX015 (a BETi) diminishes the expression of *CD274* via upregulating miR-93 and miR-106b (95). Furthermore, JQ1 in combination with anti-PD-1 antibody enhances antitumor responses in mice bearing Myc-driven lymphomas and KRAS-driven NSCLC (94).

#### Non-coding RNAs

MicroRNAs (miRs) are a group of small non-coding RNAs known to regulate target genes at the post-transcriptional level (96). Adding to the growing body of evidence, the role of miRs in regulating PD-L1 expression via several mechanisms has been demonstrated. In general, most miRs are negatively correlated with PD-L1 through inducing the degradation or translational repression of *PD-L1* mRNA by direct binding to *PD-L1* 3′-UTR, whereas several miRs are found to be positively connected with PD-L1 expression. For example, MiR-3127-5p and miR-135 are associated with elevated levels of PD-L1 in NSCLC by targeting STAT3 and TRIM16/JAK/STAT, respectively (97, 98). MiR-20b, miR-21, miR-130b, and miR-142-5P induce PD-L1 overexpression via inhibiting phosphatase and tensin homolog (PTEN) (99, 100). The detailed information for miR-mediated PD-L1 regulation is delineated in Table 2 (95, 97–128).

**Table 2:** microRNAs regulate PD-L1 expression.

**miR**	**Cell types**	**Regulation of PD-L1**	**Targets**	**References**
miR-106b	PDAC	−	−	(95)
miR-130b	CRC	+	PTEN	(99)
miR-135	NSCLC	+	TRIM16/JAK/STAT	(98)
miR-138-5p	CRC	−	3′-UTR	(101)
miR-140	NSCLC	−	3′-UTR	(102)
miR-140	Osteosarcoma	−	3′-UTR	(103)
miR-140	Cervical cancer	−	3′-UTR	(104)
miR-142	Cervical cancer	−	3′-UTR	(104)
miR-142-3p	Macrophage, DC	+	−	(105)
miR-142-5p	PDAC	−	3′-UTR	(106)
miR-142-5p	NSCLC	−	PTEN/PI3K/Akt	(100)
miR-15a	MPM	−	3′-UTR	(107)
miR-155	HDLEC & DF	−	3′-UTR	(108)
miR-16	MPM	−	3′-UTR	(107)
miR-16	Macrophage	−	3′-UTR	(109)
miR-17-5p	Melanoma	−	3′-UTR	(110)
miR-18a	Cervical cancer	+	PI3K/AKT MEK/ERK Wnt/β-catenin/STAT3 P53/miR-34a	(104)
miR-193a-3p	MPM	−	3′-UTR	(107)
miR-195	DLBCL	−	3′-UTR	(111)
miR-197	OSCC	−	−	(112)
miR-197	NSCLC	−	STAT3	(113)
miR-20b	CRC	+	PTEN	(99)
miR-200c	HCC	−	3′-UTR	(114)
miR-200c	AML	−	3′-UTR	(115)
miR-200c	NSCLC	−	3′-UTR	(116)
miR-21	Macrophage	−	JAK2/STAT1	(117)
miR-21	CRC	+	PTEN	(99)
miR-217	Laryngeal cancer	−	Translation	(118)
miR-24	Macrophage, DC	+	−	(105)
miR-30b	Macrophage, DC	+	−	(105)
miR-3127-5p	NSCLC	+	STAT3	(97)
miR-33a	LA	−	3′-UTR	(119)
miR-34	NSCLC	−	3′-UTR	(120)
miR-34a	Glioma	−	3′-UTR	(121)
miR-34a	AML	−	3′-UTR	(115, 122)
miR-340	Cervical cancer	−	3′-UTR	(104)
miR-375	HNSCC	−	JAK2/STAT1	(123)
miR-383	Cervical cancer	−	3′-UTR	(104)
miR-424(322)	Ovarian	−	3′-UTR	(124)
miR-513	Cholangiocytes	−	3′-UTR	(125, 126)
miR-513a-5p	Retinoblastoma	−	3′-UTR	(127)
miR-574-3p	Chordoma	−	−	(128)
miR-93	PDAC	−	−	(101)

*−, negative; +, positive; PDAC, pancreatic ductal adenocarcinoma; CRC, colorectal cancer; NSCLC, non-small cell lung cancer; DC, dendritic cell; MPM, malignant pleural mesothelioma; HDLEC, human dermal lymphatic endothelial cell; DF, dermal fibroblasts; DLBCL, diffuse large B-cell lymphoma; OSCC, oral squamous cell carcinoma; HCC, hepatocellular carcinoma; AML, acute myeloid leukemia; LA, lung adenocarcinoma; HNSCC, head and neck squamous cell carcinomas; PTEN, phosphatase and tensin homolog; TRIM16, tripartite-motif 16; JAK, Janus kinase; STAT, signal transducer and activator of transcription; 3′-UTR, 3′ untranslated region; PI3K, phosphatidylinositol 3 kinase; AKT, protein kinase B;MEK, MAPK/ERK kinase; ERK, extracellular regulated protein kinases; P53, protein 53*.

Moreover, the profiled miRs in serum from NSCLC patients uncover seven miRs (miR-215-5p, miR-411-3p, miR- 493-5p, miR-494-3p, miR-495-3p, miR-548j-5p, miR-93-3p) strongly associated with overall survival after treatment with the immune checkpoint inhibitor, nivolumab (129). In addition, a study in CRC highlights the role of circular RNA in accommodating PD-L1 expression, revealing that circular RNA has_circ_0020397 promotes the expression of PD-L1 by inhibiting miR-138 activity (130). More importantly, a very recent study has identified miR-146a as a comparable immune checkpoint molecule in melanoma, and a miR-146a antagomiR combined with anti-PD-1 enhances the anti-tumor effect of anti-PD-1 therapy, suggesting that miRNAs may be a novel combination target for immune checkpoint therapy (131).

### Extrinsic Control of PD-L1 Expression

#### Virus Infection and Inflammatory Signaling

Although a generalized conclusion for PD-L1 expression in virus infection remains uncertain as comparable levels of PD-L1 were detected in individuals with or without infection (74, 132–134), escalated PD-L1 levels were shown to be connected with specific viruses, such as Epstein-Barr virus (EBV) (135–139), hepatitis B viral (HBV) (114, 140, 141), hepatitis C virus (HCV) (142–146), human immunodeficiency virus (HIV) (132, 147–150), human papilloma virus (HPV) (135, 151–155), Merkel cell polyomavirus (MCPyV) (156), bovine leukemia virus (BLV) (157), and Kaposi sarcoma-associated herpes virus (KSHV) (158). The pathobiological mechanisms by which viruses trigger the expression of PD-L1 were revealed. Pathogen associated molecular patterns (PAMPs) stemming from a pathogen (for example virus, bacteria, and fungi) activate toll like receptors (TLRs) to initiate the immune response and protect the host against pathogens infection. TLR agonists-induced synthesis of PD-L1 has been observed in a variety of cell types (159–164). PAMPs from EBV was reported to induce PD-L1 upregulation in a TLR-dependent manner (136, 165, 166). Similarly, HIV stimulates PD-L1 expression on APC via TLR signaling or in an indirect manner by increasing the production of cytokines (149, 150). Several downstream signaling pathways activated by virus infection, such as JAK/STAT, MAPK, and NF-κB signaling, are involved in both TLR agonists- and virus infection-mediated regulation of PD-L1 (114, 137, 138, 159, 161, 166), further supporting the notion that TLR signaling serves as an effector for the induction of PD-L1 upon virus infection. TLRs-independent induction of PD-L1 in virus infection was also uncovered (114, 139, 148). For an example, amplification of *PD-L1* gene, a major cause of PD-L1 overexpression, was observed in EBV-positive gastric tumors (139).

Persistent infections of pathogens lead to chronic inflammation via promoting the secretion of inflammatory cytokines. Pro-inflammatory molecules or cytokines, such as IFN-γ, IFN-α, IFN-β, TNF-α, EGF, IL-17, IL-4, and IL-27 have been reported to induce PD-L1 expression in tumors (167–175). Among them, IFN-γ is the most potent inducer of PD-L1. Upregulation of PD-L1 by IFN-γ has been extensively described in diverse cell types (31, 168, 176–179). PD-L1, largely induced locally at the tumor by tumor-infiltrating lymphocytes (TILs)-derived IFN-γ, which are termed adaptive immune resistant, was first reported in melanomas by Taube et al. (180) and Abiko et al. (181). JAK/STAT and NF-κB pathways are the main downstream signals in the inflammation for IFN-γ-induced PD-L1 expression (170, 182–184). Another transcriptional mechanism is used for controlling the expression of PD-L1 by IFN-γ in melanoma and medulloblastoma, in which activation of JAK/STAT signal increases the expression of a series of transcription factors named the interferon-responsive factors (IRFs) (182, 185). Likewise, TNF-α, another pro-inflammatory cytokine, upregulates PD-L1 expression via TNF-α-NF-κB pathway (167, 170, 172, 186). Furthermore, TNF-α was reported to synergistically act with IFN-γ to induce PD-L1 expression at both mRNA and protein levels and enhance the adaptive immune resistance mediated by IFN-γ-induced PD-L1 in hepatocellular carcinoma cells via upregulating the expression of IFN-γ receptors (187).

#### Transforming Growth Factor β (TGF-β)

TGF-β, which is generally considered as an anti-inflammatory cytokine, plays a paradox role in cancer. High levels of TGF-β render tumor cells capable of escaping immune surveillance (188–190). It was recently reported that TGF-β elevated PD-L1 expression. In lung cancer, TGF-β-mediated EMT facilitates PD-L1 expression through an epigenetic mechanism (77, 191, 192). Consistently, TGF-β-induced EMT was recently revealed to promote PD-L1 expression by post-translational modification. EMT transcriptionally upregulates N-glycosyltransferase STT3 through β-catenin, and STT3 further glycosylates and stabilizes PD-L1 (193). In the mouse model of pancreatic islet transplantation, TGF-β was found to be necessary for the sustained expression of PD-L1 on CD8^+^ T cells via autocrine (194). These findings highlight that the role of TGF-β in the regulation of PD-L1 may possibly account for the link between TGF-β and immune evasion.

In addition, TGF-β shapes the tumor microenvironment to restrain antitumor immunity by restricting T cell infiltration and attenuating the efficacy of PD-L1 blockade antibody. TGF-β inhibitor and PD-L1 blockade together provoke vigorous antitumor immune response and tumor regression (189, 190). M7824, a novel bifunctional anti-PD-L1/TGF-β with a soluble extracellular domain of TGF-β receptor II, elicits potent and superior antitumor activity in preclinical and clinical studies (195–197).

#### Hypoxia

Hypoxia, an inevitable outcome due to the abnormal vasculature and a huge mass of tumor, is represented as a hallmark of a tumor microenvironment. Under the hypoxic environment, tumor cells survive themselves by reprogramming the gene expression through activating a series of hypoxia-inducible factors (HIFs). Among these HIFs, HIF-1α, and HIF-2α are the most important transcript factors responsive to hypoxia, leading to the adaption to the stress (198, 199). Hypoxia signaling represents an important pathway in immune evasion (200–202). Hypoxia markedly induces the expression of PD-L1 on the surface of myeloid-derived suppressor cells (MDSCs), macrophages, DCs, monocytes, and tumor cells (201, 203, 204). Exposure of human or murine cancer cells to hypoxia leads to the upregulation of PD-L1, which induces T cell apoptosis in a HIF-1α-dependent manner. Furthermore, blocking the accumulation of HIF-1α in hypoxic cells by glyceryl trinitrate prevents hypoxia-induced PD-L1 expression (201). In another study, inhibiting HIF-1α through gene knockdown or PX-478 treatment also strikingly attenuates the elevation of PD-L1 induced by CoCl_2_ (a hypoxia-mimic treatment) (205). Likewise, PD-L1 overexpression on monocytes is induced both *in vitro* and *in vivo* models of intermittent hypoxia, and by *HIF-1*α gene transfection (204). Moreover, HIF-1α was demonstrated to translocate into the nucleus and drive PD-L1 expression in human monocytes during endotoxin tolerance (206). Direct binding of HIF-1α to the promoter of *PD-L1* via hypoxia response element (HRE) has been unveiled in MDSCs by ChIP and luciferase reporter assay (203). Collectively, PD-L1 expression is regulated by HIF-1α in monocytes and tumor cells, including melanoma cells, breast cancer, prostate cancer, and lung cancer cells (201, 203, 204, 207). However, in clear cell renal cell carcinoma (ccRCC), cells with VHL (a component of oxygen and iron sensing pathway that regulates the HIF) mutation, HIF-2α, rather than HIF-1α, is specifically able to induce PD-L1 expression (208). The association between HIF-2α and PD-L1 was further verified in ccRCC by Messai et al. (209), where a direct binding of HIF-2α to a transcriptionally active HRE in human PD-L1 proximal promoter was revealed.

Angiogenesis in solid tumors is a multiple biological process that is induced by the overexpression of pro-angiogenic factors in the environment to support the tumor cell growth. However, in contrast to the vasculature of normal tissues, tumor vessels are usually twisted and disorganized accompanied with a reduced blood perfusion and oxygenation. Normalization of tumor vasculature with an appropriate dose of anti-angiogenic treatment, primarily through disruption of the VEGF/VEGFR axis, is able to reduce tissue hypoxia and reprogram the tumor microenvironment from immunosuppressive to immunoactive (210, 211). Remarkably, PD-L1 expression is positively correlated with VEGF and microvessel density in patients with uniformly treated cHL (212). Considering that hypoxia is capable of inducing PD-L1 expression, addition of anti-angiogenic reagent to PD-1/PD-L1 antibodies is likely to sensitize immune checkpoint therapy. This conjecture has been verified in both animals and human beings. For example, a preclinical study showed that combined anti-VEGF/anti-PD-L1 targeted therapy synergistically improves the treatment outcome, compared to both anti-PD-L1 or anti-VEGF monotherapy in an autochthonous mouse model of small cell lung cancer (SCLC) (213). Similarly, preliminary results from a phase III study (NCT02366143), which evaluated the efficacy of bevacizumab (the first anti-angiogenic drug) plus atezolizumab plus chemotherapy, bevacizumab plus chemotherapy, and atezolizumab plus chemotherapy in non-squamous NSCLC, has also revealed superior outcomes in patients receiving the first treatment (214). Choueiri et al. (215) recently reported a phase Ib trial based on avelumab plus axitinib, a VEGF receptor inhibitor as first-line therapy in patients with advanced ccRCC (NCT02493751). McDermott et al. (216) performed a phase II study of atezolizumab alone or combined with bevacizumab vs. sunitinib in treatment-naive metastatic RCC, and the preliminary data on antitumor activity are encouraging. A phase III trial assessing avelumab and axitinib compared with sunitinib monotherapy for first-line treatment of advanced RCC is ongoing (NCT02684006).

#### Previous Treatments

Radiation and conventional antineoplastics that are currently used for cancer therapy, such as chemotherapeutic drugs and epigenetic modifiers, may promote immunogenic cell death via introducing DNA damage. Importantly, it is increasingly evident that conventional radiotherapies and chemotherapies, as well as novel epigenetic modifiers and targeted anticancer agents cannot only induce tumor regression by triggering immunogenic death of tumor cells, but also result in tumor progression by dysregulating the immune system, including changes within inhibitory molecules across a wide range of malignancies. Radiation and a number of chemotherapy drugs induce PD-L1 expression through different mechanisms, which are likely responsible for the loss of antitumor immunity and acquired resistance (217, 218). Remarkably, the PD-1/PD-L1 pathway blockade reverses adaptive immune resistance and maintains the antitumor immunity (219–221). Of note, although radiation and most chemotherapies enhance PD-L1 expression upon most occasions, inconsistent results were also obtained. The detailed information and involved signal pathways are listed in Table 3 (67, 78, 113, 122, 127, 193, 217, 220–255).

**Table 3:** Alteration of PD-L1 expression by previous treatments.

**Therapeutic**	**Category/dose**	**Tumor type**	**PD-L1 expression**	**Targets**	**References**
IR	10 Gy	Breast cancer	+	−	(222)
IR	10 Gy	Breast cancer	+	IFN-γ	(223)
IR	12 Gy	Breast cancer	+	−	(224)
IR	10 Gy	CRC	+	IFN-γ	(223)
IR	4 Gy/8G y	Glioma	+	−	(225)
IR	5 Gy/10 Gy	Glioma	+	EGFR/JAK2	(226)
IR	45 Gy/60 Gy	HCC	+ (sPD-L1)	−	(227)
IR	2 Gy	HNSCC	+	AXL-PI3K	(217)
IR	10 Gy	HNSCC	+	−	(228)
IR + chemotherapy	−	HNSCC	+	IL-6/STAT3	(229)
IR	10 Gy	Lung cancer	+	ATM/ATR/Chk1 STAT1/STAT3-IRF-1	(230)
IR	6 Gy ×1 − 6 Gy ×4	NSCLC	+	IL-6-MEK/ERK	(231)
IR	10 Gy	Melanoma	+	IFN-γ	(223)
IR	10 Gy	Osteosarcoma	+	ATM/ATR/Chk1 STAT1/STAT3-IRF-1	(230)
IR	−	PDAC	+	JAK/STAT1	(232)
IR	10 Gy	Prostate cancer	+	ATM/ATR/Chk1 STAT1/STAT3-IRF-1	(230)
IR	50 - 50.4 Gy	Sarcoma	+	−	(233)
UVR	100 J/m^2^	HPKs/HPMs	+	NRF2	(234)
Arsenic trioxide	Undefined cytoxin	AML	+	MiR-34a	(122)
Azacytidine	DNMTi	NSCLC	+	−	(78)
Carboplatin	Alkylating agent	Ovarian cancer	+	JAK/STAT, antiviral defense	(235)
Carboplatin + paclitaxel	Alkylating agent + Antimicrotubule	Lung cancer	NC	−	(236)
Cisplatin	Alkylating agent	Hepatoma	+	MAPK/ERK	(237)
Cisplatin	Alkylating agent	HNSCC	+	IL-6/STAT3	(220)
Cisplatin	Alkylating agent	HNSCC	+	−	(238)
Cisplatin	Alkylating agent	HNSCC	+	MAPK/ERK	(239)
Cisplatin	Alkylating agent	Lung cancer	NC	−	(240)
Cisplatin	Alkylating agent	NSCLC	+	FASN/TGF-β1	(241)
Cisplatin	Alkylating agent	NSCLC	+	Akt, NF-κB p65	(221)
Cisplatin	Alkylating agent	NSCLC	+	PI3K/Akt	(242)
Cisplatin	Alkylating agent	SCLC	+	DNMT1,KIT	(243)
Cisplatin	Alkylating agent	NHL	+	ERK,GM-CSF	(244)
Cisplatin	Alkylating agent	Ovarian cancer	+	−	(245)
Cisplatin + gemcitabine	Alkylatingagent + Antimetabolite	Lung cancer	−	−	(236)
Decitabine	DNMTi	Myelodysplastic syndromes	+	−	(67)
Doxorubicin	Topoisomerase inhibitor	Breast cancer	− (surface) + (nuclear)	PI3K/Akt	(246)
Doxorubicin	Topoisomerase inhibitor	NHL	+	ERK,GM-CSF	(244)
Epirubicin	Topoisomerase inhibitor	Breast cancer	−	−	(247)
Etoposide	Topoisomerase inhibitor	Breast cancer	+	−	(248)
Etoposide	Topoisomerase inhibitor	Breast cancer	−	EMT/β-catenin/STT3	(193)
Etoposide	Topoisomerase inhibitor	CSCs	−	EMT/β-catenin/STT3	(193)
Etopside	Topoisomerase inhibitor	NHL	+	ERK,GM-CSF	(244)
Etoposide	Topoisomerase inhibitor	Retinoblastoma	+	MiR-513a-5p	(127)
Gemcitabine	Antimetabolite	Pancreatic cancer	+	JAK/STAT1	(232, 249)
Oxaliplatin	Alkylating agent	CRC	+	IFN-γ	(250)
Oxaliplatin	Alkylating agent	NHL	+	ERK,GM-CSF	(244)
Oxaliplatin	Alkylating agent	Prostate cancer	+	TGF-β	(251)
Paclitaxel	Antimicrotubule	Breast cancer	+	−	(247, 248)
Paclitaxel	Antimicrotubule	CRC	+	ERK1/2	(251)
Paclitaxel	Antimicrotubule	HCC	+	ERK1/2	(251)
Paclitaxel	Antimicrotubule	Ovarian cancer	+	NF-κB	(252)
Paclitaxel	Antimicrotubule	Pancreatic cancer	+	JAK2/STAT1	(249)
Platinum	Alkylating agent	NSCLC	+	MiR-197/CKSIB/STAT3	(113)
Trabectedin	Undefined cytoxin	Ovarian cancer	+	IFN-γ	(253)
Vincristine	Alkaloid	NHL	+	ERK,GM-CSF	(244)
Vinorelbine tubulin inhibitor	Antimicrotubule	Lung cancer	−	EMT	(240)
5-Fluorouracil	Antimetabolite	Breast cancer	+	−	(248)
5-Fluorouracil	Antimetabolite	CRC	+	−	(254)
5-Fluorouracil	Antimetabolite	Esophageal adenocarcinoma	+	−	(254)
5-fluorouracil	Antimetabolite	Pancreatic cancer	+	JAK2/STAT1	(249)

*−, negative; +, positive; NC, not change; IR, irradiation; UVR, ultraviolet radiation; CRC, colorectal cancer; HCC, hepatocellular carcinoma; HNSCC, head and neck squamous cell carcinomas; NSCLC, non-small cell lung cancer; PDAC, pancreatic ductal adenocarcinoma; HPKs, human primary keratinocytes; HPMs, human primary melanocytes; AML, acute myeloid leukemia; SCLC, small cell lung cancer; NHL, non-Hodgkin's lymphoma; CSCs, cancer stem-like cells; sPD-L1, soluable programmed death ligand-1; IFN-γ, interferon-γ; EGFR, epidermal growth factor receptor; JAK2, Janus kinase 2; AXL, tyrosine-protein kinase receptor UFO;IL-6, interleukin-6; STAT3, signal transducer and activator of transcription 3; ATM, ataxia telangiectasia mutated; ATR, ataxia telangiectasia and Rad3-related protein; IRF-1, interferon-responsive factor 1; NRF2, nuclear factor E2-related transcription factor 2;MAPK, mitogen-activated protein kinase; ERK, extracellular signal-regulated kinase; EMT, epithelial-mesenchymal transition; FASN, fatty acid synthase; TGFβ1, transforming growth factor β1; Akt, protein kinase B; NF-κB, nuclear factor kappa B; PI3K, phosphatidylinositol 3 kinase; DNMT1, DNA methyltransferase 1; KIT, receptor tyrosine kinase; GM-CSF, granulocyte macrophage colony-stimulating factor; CKS1B, CDC28 protein kinase regulatory subunit 1B*.

In addition to the genotoxicity therapy aforementioned, the correlations between PD-L1 expression and target therapy, as well as immunotherapy, were also documented. For example, epidermal growth factor receptor (EGFR), a commonly mutated oncogene in NSCLC, was reported to be associated with PD-L1 upregulation (256). Treatment with EGFR tyrosine kinase inhibitors (EGFR-TKIs) results in the downregulation of PD-L1 (240, 256, 257). In parallel, application of BRAF and MEK inhibitors is also associated with decreased PD-L1 expression (258). Interestingly, PD-L1 expression is upregulated through both JUN and STAT3 after acquiring resistance to BRAF inhibition in the cells (258, 259), and hence, the expression level of PD-L1 may serve as a biomarker for predicting the probability of response to the inhibitors (260). Different from EGFR and BRAF inhibitors, sorafenib, a multi-target antitumor drug, increases PD-L1 expression through inducing tumor hypoxia (261). Furthermore, several immunotherapies were found to upregulate PD-L1 expression (262, 263). Rice et al. (262) revealed an increase in PD-L1 expression on tumor cells during an HPV-E6/E7 immunotherapy. While the increased expression of PD-L1 is blunted and even reversed when combined with anti-PD-1 antibody, as a reduction in tumor PD-L1 expression was observed. These observations imply a role of anti-PD-1 therapy for regulating PD-L1. What's more, the dynamic change of PD-L1 expression on circulating tumor cells in advanced solid tumor patients undergoing PD-1 blockade therapy might serve as a predictor to indicate the therapeutic response at an early time (264). Collectively, these studies may open up new avenues for developing rational combination of cancer therapeutics in various solid tumors.

### Intrinsic Control of PD-L1 Expression

Abnormal signal transductions induced by intrinsic oncogenic activation or loss of tumor suppressor can both regulate PD-L1 expression at various levels.

#### Oncogenic Signaling

Apart from the reports described above, the intrinsic cellular changes associated with PD-L1 expression have attracted much attention because such explorations can not only expand the investigation in regulatory mechanism of PD-L1, but also direct the concomitant use of immune checkpoint therapies and target therapies for optimizing clinical outcomes. Oncogenic signaling stemming from aberrant transcription factors, effectors and upstream receptors can regulate the expression of PD-L1.

A number of oncogenic transcription factors, such as MYC, AP-1, STAT, IRF1, HIF, and NF-κB, were reported to individually or cooperatively promote PD-L1 expression at the transcriptional level. MYC, a transcription factor governing a large number of gene expressions, plays a vital role in tumorigenesis through its multiple effects on tumor cells, typically by controlling cell proliferation and survival, and elevated expression of MYC was found in approximately 70%of human cancers (265). A recent study by Casey et al. (266) revealed a novel role of MYC in cancer immunosurveillance. MYC binds to the *PD-L1* promoter transcriptionally regulating PD-L1 expression. Similarly, transient transfection with MYC plasmids upregulates PD-L1 in anaplastic large-cell lymphoma (ALCL) cells with low endogenous PD-L1 (267). In line with these observations, both pharmacological inhibition and geneticsilencing of MYC reduce PD-L1 expression in tumor cells (266–268). Moreover, a very recent study revealed a translational mechanism of MYC-mediated PD-L1 upregulation by bypassing the repressive effect of non-canonical upstream open reading frames in the 5′ untranslated region of PD-L1 on its translation (269). Of note, the translational inhibitor, eFT508, possesses a potent inhibitive effect on the cancer progression and metastasis by targeting PD-L1 mRNA translation, disclosing a new strategy for immunotherapy in PD-1/PD-L1 axis blocking.

AP-1 is a dimeric transcription factor comprised of Jun, Fos, and ATF protein families (270). As the best-known member of the AP-1 family, c-Jun is implicated in PD-L1 expression. An increased expression of PD-L1 was found in BRAF inhibitor-resistant melanoma cells. Knockdown of c-Jun results in a reduction of PD-L1 expression (259). AP-1 binding sites were identified in the first intron of *PD-L1*, and c-Jun and JUNB have been shown to be recruited to the *PD-L1* promoter (138, 271). Co-activation of STAT3 with c-Jun further enhances the transcriptional activity of PD-L1 (272, 273). Accordantly, concurrent knockdown of STAT3 and c-Jun contributes to a synergistic downregulation of PD-L1 (259). Not surprisingly, activated STAT3 alone can also increase PD-L1 expression by directly acting on the promoter of *PD-L1* in HNSCC and lymphoma cells (267, 274, 275), and STAT3 silencing leads to the downregulation of PD-L1 in ALK-negative ALCL cells and KRAS-mutant NSCLC cells (267, 271). As previously described, STAT1 and IRF1/7, downstream effectors of STAT1, can also induce PD-L1 expression (79, 169, 276). Similarly, HIF-1α and HIF-2α transcriptionally facilitate PD-L1 expression by binding to the HRE of its promoter (203, 209). Moreover, the interaction between STAT3 and HIF-1 involves in the regulation of HIF target genes. Inhibition of STAT3 decreases the expression of HIF-1 target genes in MDA-MB-231 and RCC4 cells (277), indicating a potentially cooperative effect for STAT3 and HIF-1 in regulating PD-L1. Binding of the RELA/p65-MUC1-C complex on the promoter of *PD-L1* was observed in NSCLC (278), suggesting that NF-κB may directly regulate PD-L1 transcription. Besides the above transcription factors, recent work have verified nuclear factor E2-related transcription factor 2 (NRF2) as an upstream transcriptional activator of PD-L1 in human primary keratinocytes and melanocytes after ultraviolet-B irradiation, and depletion of NRF2 significantly increases T cells infiltration in the tumors and suppresses melanoma progression (234).

Oncogenic signaling initiated by activating mutations or amplification in receptor tyrosine kinases plays an important role in regulating PD-L1. The MEK-ERK pathway is commonly activated by mutations in RAS GTPase, BRAF, and EGFR (279), and there are now abundant data suggesting that MEK-ERK signaling upregulates PD-L1 expression. Coelho et al. (280) revealed that RAS stabilizes *PD-L1* mRNA and upregulates cell-intrinsic PD-L1 expression via phosphorylation and inhibition of the adenylate-uridylate-rich element-binding protein, tristetraprolin, in a p38 MAPK-dependent manner. Furthermore, MAPK signaling is responsible for the increased expression of PD-L1, since inhibition of MEK or ERK partially offset the ectopic expression of PD-L1 in both mouse and human KRAS-mutant lung cancer cells (271, 280, 281). However, in contrast to the positive relationship between MEK-ERK signaling and PD-L1 expression observed in most studies, MEK inhibitors are unable to change the PD-L1 expression in melanoma (282), and even increase the levels of PD-L1 in breast cancer and NSCLC cells (283, 284). Similarly, in BRAF mutation melanoma cells, which acquire resistance to BRAF inhibition, constitutive PD-L1 is elevated through cooperative activation of Jun (a primary target of MAPK signaling) and STAT3 (259). Induction of PD-L1 is dependent on MAPK activation in EGFR mutant NSCLC. Suppression of ERK1/2/c-Jun results in reduced PD-L1 expression (285). Evidences also imply the role of p65, AKT/STAT3, and JAK2/STAT1 as mediators in the regulation PD-L1 expression by EGFR signaling (168, 286, 287). Moreover, the increased PD-L1 expression upon EGFR activation is also mediated by the AKT-mTOR pathway, inhibition of which (with rapamycin) abolishes the increased expression of PD-L1 upon EGFR activation through increasing lysosomal protein degradation (174). Remarkably, although EGFR activation was shown to increase PD-L1 expression through multiple signal pathways, a controversial correlation between EGFR activation mutations and PD-L1 expression was observed in NSCLC patients (256, 288–290), which may be attributed to the complicated regulation of PD-L1 expression (inducible and constitutive) *in vivo*. Therefore, despite how EGFR mutations upregulate the constitutive expression of PD-L1 in tumor cells, the concomitant shortage of activated tumor infiltrating lymphocytes in EGFR-mutated NSCLC (291, 292) may weaken the inducible PD-L1 expression. Recent studies have shielded light on the divergent effect of various subtypes of EGFR mutations on PD-L1 expression and the response to immune checkpoint therapy (293, 294). Thus, the discrepancies in PD-L1 expression caused by EGFR mutations may also result from the undistinguished subtypes of EGFR mutations.

The PI3K-AKT-mTOR signaling is another oncogenic pathway involved in constitutive regulation of PD-L1, which can both be activated by *PIK3CA* mutation and functional loss of PTEN (a negative regulatory of PI3K-AKT signaling) (295). In human glioma and CRC cells, loss of PTEN activates PI3K signaling, which leads to an elevation of PD-L1 expression (296, 297). A positive correlation between *PIK3CA* mutation and PD-L1 expression in squamous cell lung carcinoma was also revealed (298). Nevertheless, similar to the MEK inhibition, the influence of PI3K/AKT inhibition on PD-L1 expression varies in tumor cells and the mechanism is largely unknown (299). Phosphorylation activation of S6K1 or inhibition of eIF4E-binding proteins (4E-BP, a negative regulator for eIF4E), which serve as the downstream effect for the activation of PI3K-AKT-mTOR pathway, are involved in the promotion of protein synthesis. Overexpression of S6K1, but not eIF4E, restores the translational efficiency of PD-L1, which is inhibited by ectopic expression of PTEN in U87 cells (a glioma cell lines with no PTEN expressed) through increased recruitment of PD-L1 transcript to the polysome. In line with this finding, rapamycin, an mTOR inhibitor, was shown to pose as an obstacle for polysomal component recruitment in PD-L1 transcripts (296). Interestingly, in a recent study carried out by Cerezo et al. (299), inhibition of eIF4F complex (consisting eIF4A, eIF4E, and eIF4G subunits) was shown to upregulate IFN-γ-induced PD-L1 expression by thwarting the translation of *STAT1* mRNA in melanoma, and the upstream regulator for the signaling remains to be identified. Additionally, NPM-ALK or EML4-ALK fusion protein constitutively activates ALK kinase and promotes PD-L1 expression via MEK-ERK and PI3K-AKT signaling pathways (300, 301).

Yes-associated protein/WW domain-containing transcription regulator 1 (YAP/TAZ), the well-known effectors in the Hippo pathway, are commonly dysregulated in cancers (302). Overexpression of YAP/TAZ has been found in many cancers due to abnormal amplification, loss of Hippo signaling by mutation, and/or downregulation of the core Hippo component (303). Recent publications supported the notion that YAP/TAZ emerges as a pivotal player in tumor immunity by regulating PD-L1 expression. YAP/TAZ interacting with the *PD-L1* promoter through the TEA domain transcription factor (TEAD) family of transcription factors enhances PD-L1 at the transcriptional level in human malignant pleural mesothelioma, melanoma, breast and lung cancer cells (304–309). Furthermore, in tumor tissues of NSCLC and melanoma, immunohistochemistry showed significantly positive staining for YAP and PD-L1 (305, 307). The role of the Hippo pathway in upregulating PD-L1 was further confirmed in breast and lung cancer cells as mammalian STE20-like kinase 1 and 2 (MST1/2) and large tumor suppressor 1 and 2 (LATS1/2), upstream kinases and inhibitors of canonical Hippo pathway are shown to suppress PD-L1 expression (306).

#### The Role of Tumor Suppressors

In addition to the involvement of oncogenes in the regulation of PD-L1, tumor suppressors also play a role in controlling PD-L1 expression. *Tp53*, also known as p53, is a well-known tumor suppressor and commonly mutated in cancer (310). As a transcription factor, p53 regulates the expression of numerous downstream target genes involved in cell cycle progression, cell death, and metabolism (311, 312). Increasing evidence for p53 in regulating immune responses made it intriguing for researchers (313, 314). P53 regulates immune responses by targeting immune checkpoints, including PD-L1. The effect of p53 on PD-L1 expression is likely to be mediated by several p53-regulated miRNAs. Cortez et al. (120) revealed that p53 decreases PD-L1 expression via upregulating miR-34 in NSCLC. A recent study showed that the miR-200 family, another miRNA cluster regulated by p53 (315), downregulates PD-L1 by directly targeting 3′-UTR in HCC, AML and NSCLC cells (114–116). In addition, p53 and PD-L1 expression are inversely correlated in hepatocellular carcinoma and NSCLC patients (120, 316). Coincidently, inactive mutation of p53 is associated with elevated PD-L1 level in lung adenocarcinoma and ovarian cancer (317–319). However, paradoxical results were also found by researchers, indicating an ambivalent role of p53 in PD-L1 regulation. MiR-18a increases PD-L1 levels by targeting SOX6 (p53 pathway activator) to inhibit p53 signaling in cervical cancer (104), and nutlin-3a, a small molecule activator of wild-type p53, enhances the expression of PD-L1 in breast cancer (320). Moreover, a high rate of PD-L1 expression was observed in p53-positive primary pulmonary lymphoepithelioma-like carcinoma patients, compared to the p53-negative group (321).

Tumor suppressor gene *PTEN* is one of the most frequently mutated genes in human cancers (322). PTEN acts as a tumor suppressor through the action of its phosphatase protein product, which catalytically dephosphorylates phosphatidylinositol(3,4,5)-trisphosphate (PIP3) converted to phosphatidylinositol(4,5)bisphosphate (PIP2) (323). The enzymatic activity of PTEN further modulates PD-L1 expression through the PI3K/AKT pathway (174, 296, 297, 324). In these studies, loss or knockdown of PTEN leads to the activation of the PI3K/AKT pathway, and hence, upregulation of PD-L1. In glioma, loss of PTEN function upregulates PD-L1 expression at the translational level through AKT-mTOR-S6K1 signal axis (296). In CRC, miR-20b, miR-21, and miR-130b inhibit PTEN, resulting in PD-L1 overexpression (99). A similar effect of miR-18a was revealed in cervical cancer (104). In contrast to the findings in CRC and cervical cancer, miR-142-5p was reported to promote antitumor immunity in NSCLC by suppressing PD-L1 protein expression via the PTEN pathway (100). Loss of PTEN cytoplasmic expression is related to lower PD-L1 expression in DLBCL with AKT hyperactivation (325), suggesting that mechanisms unrelated to AKT may also be involved in PD-L1 expression in cells with different PTEN status.

Strikingly, concurrent *TP53* and *PTEN* deletion facilitates the formation of undifferentiated pleomorphic sarcomas, with elevated PD-L1 expression, that induced immune tolerance in C57BL/6J mice (326). Simultaneous loss of *PTEN* and *LKB1* (a tumor suppressor, also known as serine-threonine kinase 11) contributes to the development of murine lung squamous cell carcinoma with higher PD-L1 expression, while PD-L1 in the tumor is not induced by individual deficiency of either *PTEN* or *LKB1* (327). Likewise, concomitant knockout of Kelch-like ECH-associated protein 1 (*KEAP1*) and *PTEN* in the mouse lung promotes the occurrence of adenocarcinoma with altered immune microenvironment exhibiting increased expression of PD-L1 (328). Tumor suppressor candidate 2 (TUSC2, also known as FUS1) represents another suppressor in controlling PD-L1 by recent work. TUSC2 overexpression was observed to decrease PD-L1 expression and associate with an immunologic response (329, 330). TUSC2 was shown to negate the kinase activity of EGFR, AKT, and mTOR according to previous studies (331, 332). Indeed, the reduction of PD-L1 expression in NSCLC cell lines by TUSC2 is likely due to reduced mTOR activity (330).

The retinoblastoma protein (RB), a well-acknowledged tumor suppressor, acts as a multifunctional protein to regulate various aspects of cellular activities and tumor development (333, 334). Transcription factor E2F1 is a major target for RB to exhibit its tumor suppression efficacy, un- or hypo-phosphorylated RB binds to E2F1, leading to the inhibited expression of E2F1 target genes and arrested G1/S cell cycle transition. However, during a normal cell cycle, RB transforms into hyperphosphorylated status, mediated by cyclin/CDK complexes such as CYCLIN D/CDK4/6 in the late G1 phase, which contributes to the release of E2F1 and advance of the cell cycle (335). Interestingly, an E2F1-independent tumor suppression effect of phosphorylated RB has been unveiled by Jin et al. (336), and it is said that CDK4/6 mediated phosphorylation of RB at S249/T252 enhances its interaction with NF-κB p65 within nucleus and reduces the expression of NF-κB target genes, including PD-L1. RL-S249/T252D, a small phospho-mimetic peptide of RB, is able to inhibit tumor growth in immune-proficient mice, and the regression of tumor may be partly attributed to the blocked expression of PD-L1 by RL-S249/T252D. Finally, mutations of the breast cancer type 1 and 2 susceptibility (*BRCA1/2*) genes, that are associated with familial breast cancer and ovarian cancer, were documented to be involved in immune dysfunction (337, 338).The observation of increased expression of PD-L1 in *BRCA1/2*-mutated ovarian cancer implies a possible role of BRCA1/2 for PD-L1 regulation, although the underlying mechanism remains to be explored (319, 338, 339).

### The Role of Post-translational Modifications in PD-L1 Expression

PD-L1 protein can be phosphorylated, ubiquitinated, and glycosylated after translation. These post-translational modifications (PTMs) collaboratively or competitively regulate the level of PD-L1 in cells.

The potential phosphorylation sites of PD-L1 have been predicted using the PhosphoSite database (PhosphoSite Plus Protein Page: Pd-L1 Human, 2018). However, in spite of this, the phosphorylation of PD-L1 has been sparsely reported. Horita et al. (340) identified atyrosine phosphorylation of PD-L1 induced by EGF, through a set of high-affinity and high-specificity post-translational modification enrichment tools, with no specific mechanism or mediator described. Only very recently, studies have started to shine light on the regulators for phosphorylation modification of PD-L1. Glycogen synthase kinase 3β (GSK3β) was shown to phosphorylate PD-L1 and an evolutionarily conserved GSK3β phosphorylation motif (S/TXXXS/T) at T180 and S184 was found in *PD-L1* (173). Another serine/threonine protein kinase AMP-activated protein kinase (AMPK), a sensor of cellular energy, was latterly revealed to directly phosphorylate PD-L1 at S195 (341). Both AMPK- and GSK3β-mediated phosphorylation of PD-L1 reduce the expression of PD-L1 via increasing its degradation, and disparate mechanisms were exploited to this end. Phosphorylation of PD-L1 at S195 by AMPK activation induces abnormal endoplasmic reticulum (ER) mannose trimming and produces aberrant glycoprotein of PD-L1 with mannose-rich glycan structures, which triggers the ER-associated degradation of PD-L1 (341). In contrast, GSK3β-mediated phosphorylation often facilitates ubiquitin E3 ligase recognition, which targets proteins to proteasomal degradation (342, 343). Thus, GSK3β phosphorylates non-glycosylated PD-L1 and further initiates the interaction of PD-L1 with β-TrCP to form a complex, leading to the poly-ubiquitination of PD-L1. Furthermore, enhanced PD-L1 expression, induced by EGF stimulation, may be attributed to its effect on inhibiting GSK3β-mediated phosphorylation and poly-ubiquitination of non-glycosylated PD-L1 (173). EGF is also involved in upregulating the ubiquitination of glycosylated PD-L1, and the ubiquitination of glycosylated PD-L1 is identified to be mono- and multi-ubiquitnation, but not poly-ubiquination (340). In contrast to GSK3β induced poly-ubiquitination of non-glycosylated PD-L1, that promotes its degradation via the proteasome pathway, EGF-stimulated mono- and multi-ubiquitination of glycosylated PD-L1 has yielded PD-L1 overexpression, as blocking the enzyme activity of ubiquitin E1 decreases PD-L1 mono- and multi-ubiquitination coupled with reduced PD-L1 level (340). The cooperative regulation of non-glycosylated and glycosylated PD-L1 induced by EGF may collaboratively increase the level of PD-L1. In a recent study, ubiqutin E3 Cbl-b/c-Cbl was revealed to be negatively correlated with PD-L1 expression in EGFR wild-type NSCLC (344). Similarly, in cervical and breast cancer cells, cyclin D1-CDK4 kinase was reported to destabilize PD-L1 and increase TILs via phosphorylating cullin 3-speckle type POZ protein (CUL3^SPOP^) E3 ligase, leading to the ubiquitination of PD-L1 (345, 346). DCUN1D1, a regulator of ubiquitin E3 activity, was significantly increased in NSCLC tumor tissues and positively associated with PD-L1 expression, which leads to enhanced tumor metastasis and poor prognosis of the patients (347). Moreover, a ubiquitously expressed type-3 transmembrane protein, CKLF-like MARVEL transmembrane domain containing protein 6 (CMTM6), was recently identified as a novel positive regulator of PD-L1 via increasing its half-life, which is presumably due to the reduction of ubiquitination and prevention of lysosome-mediated degradation during protein recycling (348–350). Notably, this function is shared by its closest family member, CMTM4 (349). In CRC, huntingtin-interacting protein 1-related protein (HIP1R), a newly discovered regulator of PD-L1, was found to promote lysosome-mediated degradation of PD-L1 by directly binding and transporting it to lysosome via the lysosomal sorting motif of HIP1R (351). Finally, in addition to the transcriptional regulation of PD-L1, TNF-α-mediated nuclear translocation and downstream transactivation of p65 increase PD-L1 expression by transcriptionally upregulating CSN5, which functionally hydrolyzes the ubiquitin chain from ubiquitin-PD-L1 and further enhances the PD-L1 stabilization in breast cancer cells (167, 186).

PD-L1 is a ~33 kDa type-1 transmembrane protein. However, detection of PD-L1 by immunoblotting revealed heterogeneous expression patterns on SDS-PAGE, and the high molecular weight of PD-L1 displays ~15-kDa molecular weight shift down by glycosylation inhibitors, suggesting that PD-L1 is highly glycosylated in human tumor tissues and cancer cell lines. Further studies identified that the type of glycosylation is primarily N-glycosylation rather than O-glycosylation since glycosylation of PD-L1 was completely inhibited by N-linked glycosylation inhibitor tunicamycin (TM) but not O-glycosidase (173). N-glycosylation occurred on the asparagine residue of an Asn-X-Ser/Thr motif (X is any amino acid except proline) in protein and is catalyzed by oligosaccharyltransferase, acting as a biosynthetic secretory pathway in ER and Golgi apparatus (352, 353). Amino-acid sequence comparation across different species identifies four evolutionarily conserved NXT motifs (N refers to Asn) in PD-L1 extracelluar domain and exclusive N-glycosylation of PD-L1 at N35, N192, N200, and N219 is further demonstrated by mass spectrometry and mutagenesis assays. Furthermore, N-glycosylations on the N192, N200, and N219 of PD-L1 cause a spatial hindrance for its interplay with GSK3β and competitively antagonize the GSK3β-mediated phosphorylation of PD-L1, and thus contribute to PD-L1 protein stability. Additionally, glycosylation of PD-L1 appears to affect its interaction with PD-1 and T-cell-mediated cytolysis (173, 354). Increasing in the affinity for PD-1 to glycosylated PD-L1 was also corroborated in another study (355). FKBP51s, a spliced isoform of 51 KDa FK506-binding protein (FKBP51), which is a cochaperone and plays a role in immunoregulation and basic cellular processes involved in protein folding and trafficking, was shown to physically interact with the naïve PD-L1 in the ER and catalyze PD-L1 folding, thus contribute to the glycosylation of PD-L1 in glioma as proposed by D'Arrigo et al. (225). Consistently, silencing of FKBP51s significantly reduces the level of glycosylated PD-L1 (225). Latterly, an emerging report by Maher et al. (356) demonstrated a physical association between multifunctional chaperone/scaffolding protein sigma1 and early formed glycosylated PD-L1 in the ER in triple-negative breast cancer (TNBC) and prostate cancer cells. The co-occurrence of reduced PD-L1 expression in both the intracellular membrane and plasma membrane induced by sigma1 inhibition indicates that both protein stability and trafficking are involved in the regulation of PD-L1 by sigma1 (356). Collectively, stabilization of PD-L1 by FKBP51s and sigma1 are both related to the glycosylation process in the ER (225, 356). Besides, dysregulation of PD-L1 glycosylation in the ER induced by AMPK activation decreases the stability of PD-L1 and triggers its ER-associated degradation (341). Furthermore, evidence has also been obtained for a possible link of EMT to PD-L1 stabilization through glycosylation regulation. EMT transcriptionally upregulates the N-glycosyltransferase STT3 through β-catenin, and subsequently induces STT3-dependent PD-L1 N-glycosylation (193). Strikingly, the latest results have proven that glycosylated PD-L1 could serve as valuable therapeutic target for cancer (341, 354, 357). Eradication of triple-negative breast cancer cells can be achieved by treatment with STM108-MMAE, a drug-conjugated antibody specifically targeted to glycosylated PD-L1, via promoting PD-L1 internalization and lysosomal degradation (354, 357). Owing to the aberrant glycosylation of PD-L1 in the ER, mediated by metformin, the combination of anti-CTLA4 antibody and metformin successfully invigorates a potent anti-cancer effect and enhances tumor elimination compared to the monotherapy in a syngeneic mouse model of melanoma, breast, and colon cancer (341).

Besides the post-translational modifications mentioned above, protein lipidations, such as prenylation, myristylation, and palmitoylation, are another prevalent type of post-translational modification. Among them, palmitoylation (also known as S-acylation) is quite different due to its reversible feature and may serve as a therapeutic target for diseases, including tumor (358). Palmitoylation is proven to play a pivotal role in regulating protein traffic, membrane localization, and interaction (359). Palmitoylated proteins also display altered protein stability and signal transduction (358–360). Since the discovery of palmitoylation modification of proteins, it has been revealed that nearly 1,000 proteins can be palmitoylated in human beings (361). Intriguingly, a single palmitoylation site at Cys272 of PD-L1, which is located in cytosolic domain, has been unveiled based on online prediction (csspalm.biocuckoo.org) and further verified by acyl-biotin exchange assays combined with mutation (362). Palmitoylation of PD-L1 in tumor cells contributes to its increased stability and avoidance of immune surveillance. It is believed that palmitoylation can spontaneously occur *in vitro*, and *in vivo*, it is usually catalyzed by protein acyltransferases (PATs). Although palmitoylation has been discovered for a long time, related PATs were identified much later (363, 364). Most PATs that mediated protein palmitoylation belong to the zinc finger protein family and possess a conserved Asp-His-His-Cys (DHHC) domain (364). In the study by Yang et al. (362), palmitoyltransferase ZDHHC9 was demonstrated to be responsible for the palmitoylation of PD-L1. The interaction between ZDHHC9 and PD-L1 is affirmed, knockdown of ZDHHC9 in cancer cells induces a significant reduction of PD-L1 palmitoylation and sensitizes T-cell-mediated killing, hence inhibiting tumor growth. This study raises a possible approach for targeting PD-L1 palmitoylation to restore the immune surveillance and cytolytic activity of T cells for cancer treatment. This was further demonstrated by Yao et al. recently (365). In contrast to the findings in breast cancer, Yao et al. have identified palmitoyltransferase ZDHHC3 (DHHC3) as the main acyltransferase required for the palmitoylation of PD-L1 in CRC cells, inhibition of which by 2-bromopalmitate and a synthetic peptide successfully decreases PD-L1 expression and enhances T-cell immunity against the tumors.

PTMs may affect the conformation, activity, and interactions of proteins. Although the PTMs, e.g., glycosylation, phosphorylation, ubiquitination, and palmitoylation of PD-L1, have been reported to influence its expression, the possible effects of these PTMs on the conformation and molecular interactions of PD-L1/PD-1 remain rather limited. PD-L1 can also be acetylated and SUMOylated in response to EGF (340). Thus, to fully understand the role of PTMs in PD-L1 regulation, more studies are needed to (1) characterize the types of PTMs of PD-L1, (2) decode the modification sites and the functional consequences of PTMs, (3) clarify the interactions among these PTMs, (4) evaluate the availability of PD-L1 PTMs as potential targets in PD-1/PD-L1 axis for cancer treatment.

**Figure 1:**
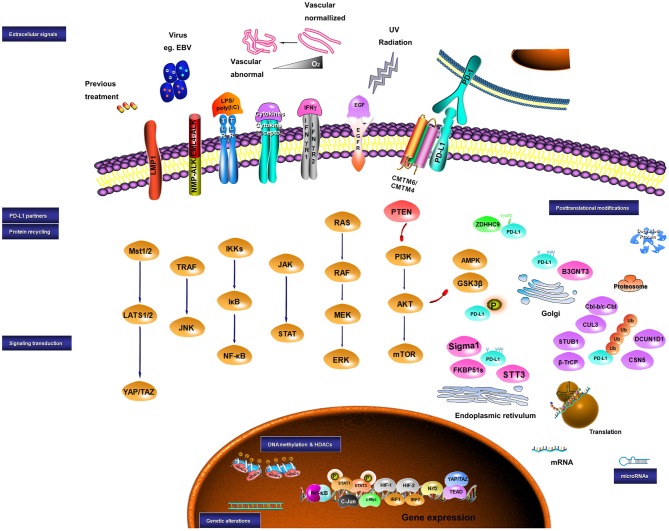
Sketch diagram for regulatory mechanisms in PD-L1 expression. Multiple factors are involved in the regulation of PD-L1 at different levels. The intrinsic and extrinsic signals implicating the regulation of PD-L1 are presented.

Collectively, the expression of PD-L1 is controlled by both intrinsic and extrinsic signaling, which may share similar molecular mechanisms. Crosstalk among these signaling pathways also plays a role in PD-L1 expression (184, 366). In summary, the level of PD-L1 in a tumor can be modulated by the genomic aberrances, epigenetic alterations, and extracellular stimuli in a very complex way, which may mechanistically work through transcriptional control, mRNA stability, oncogenic signaling pathway, and protein stability (Figure 1).

## Summary

Despite considerable improvement of cancer therapy, which has been achieved through PD-1/PD-L1 blockade, the knowledge regarding the biology of these regulators in cancer immune surveillance is still relatively limited. Many mechanisms have been revealed to regulate the expression of PD-L1 including genetic alterations, epigenetic modifiers, extracellular stimulations, signaling pathways, transcriptional factors, and post-transcriptional modulators. Generally, PD-L1 on tumor cells is regulated with two patterns: inducible and constitutive expression. Inducible expression of PD-L1 by the inflamed microenvironment within a tumor or by previous treatments may portend a better response to anti-PD-1/PD-L1 therapies and provide an opportunity for overcoming acquired resistance to prior treatments. Uncovering the mechanisms of constitutive PD-L1 expression driven by oncogenic signaling is valuable for developing new strategies for cancer therapy through directly targeting PD-L1 (269, 299). Similarly, synthetic peptides that either target PD-L1 degradation or post-translational modifications have shown a strong efficiency in animal studies, which may serve as a novel therapy for cancer treatment (351, 365). Notably, this kind of therapy that directly targets PD-L1 is of great significance and may be more effective as compared with blockade of PD-1/PD-L1 axis due to PD-1-independent functions of PD-L1 in the promotion of malignant phenotypes and drug resistance (367, 368). Altogether, an extensive understanding of the mechanisms by which PD-L1 is governed will help us to reach a comprehensive evaluation of PD-1/PD-L1 targeting therapy, and further potentiate the efficacy and expand the usage of this kind of cancer therapy via patient selection and rational combination with other antineoplastic agents, as well as develop new effective strategies for cancer immunotherapy.

## Author Contributions

XS wrote the manuscript. Z-XX contributed to the conception and writing. JL drew the tables and created the figure in the manuscript. LZ, YL, and YW revised the manuscript. All authors read and approved the final manuscript.

### Conflict of Interest Statement

The authors declare that the research was conducted in the absence of any commercial or financial relationships that could be construed as a potential conflict of interest.

## References

[1] Robert C Long GV Brady B Dutriaux C Maio M Mortier L. Nivolumab in previously untreated melanoma without BRAF mutation. N Engl J Med. (2015) 372:320–30. 10.1056/NEJMoa1412082 25399552

[2] Weber JS D'Angelo SP Minor D Hodi FS Gutzmer R Neyns B. Nivolumab versus chemotherapy in patients with advanced melanoma who progressed after anti-CTLA-4 treatment (CheckMate 037): a randomised, controlled, open-label, phase 3 trial. Lancet Oncol. (2015) 16:375–84. 10.1016/S1470-2045(15)70076-8 25795410

[3] Garon EB Rizvi NA Hui R Leighl N Balmanoukian AS Eder JP. Pembrolizumab for the treatment of non-small-cell lung cancer. N Engl J Med. (2015) 372:2018–28. 10.1056/NEJMoa1501824 25891174

[4] Antonia SJ Villegas A Daniel D Vicente D Murakami S Hui R. Durvalumab after chemoradiotherapy in stage III non-small-cell lung cancer. N Engl J Med. (2017) 377:1919–29. 10.1056/NEJMoa1709937 28885881

[5] Fehrenbacher L von Pawel J Park K Rittmeyer A Gandara DR Ponce Aix S Updated efficacy analysis including secondary population results for OAK: a randomized phase III study of atezolizumab vs docetaxel in patients with previously treated advanced non-small cell lung cancer. J Thorac Oncol. (2018) 13:1156–70. 10.1016/j.jtho.2018.04.039 29777823

[6] Motzer RJ Rini BI McDermott DF Redman BG Kuzel TM Harrison MR. Nivolumab for metastatic renal cell carcinoma: results of a randomized phase II trial. J Clin Oncol. (2015) 33:1430–7. 10.1200/JCO.2014.59.0703 25452452 PMC4806782

[7] Meti N Esfahani K Johnson NA. The role of immune checkpoint inhibitors in classical Hodgkin lymphoma. Cancers. (2018) 10:E204. 10.3390/cancers10060204 29914088 PMC6025119

[8] Ansell SM Lesokhin AM Borrello I Halwani A Scott EC Gutierrez M PD-1 blockade with nivolumab in relapsed or refractory Hodgkin's lymphoma. N Engl J Med. (2015) 372:311–9. 10.1056/NEJMoa1411087 25482239 PMC4348009

[9] Powles T Eder JP Fine GD Braiteh FS Loriot Y Cruz C. MPDL3280A (anti-PD-L1) treatment leads to clinical activity in metastatic bladder cancer. Nature. (2014) 515:558–62. 10.1038/nature13904 25428503

[10] Plimack ER Bellmunt J Gupta S Berger R Chow LQ Juco J. Safety and activity of pembrolizumab in patients with locally advanced or metastatic urothelial cancer (KEYNOTE-012): a non-randomised, open-label, phase 1b study. Lancet Oncol. (2017) 18:212–20. 10.1016/S1470-2045(17)30007-4 28081914

[11] Larkins E Blumenthal GM Yuan W He K Sridhara R Subramaniam S FDA approval summary: pembrolizumab for the treatment of recurrent or metastatic head and neck squamous cell carcinoma with disease progression on or after platinum-containing chemotherapy. Oncologist. (2017) 22:873–8. 10.1634/theoncologist.2016-0496 28533473 PMC5507654

[12] Chow LQM Haddad R Gupta S Mahipal A Mehra R Tahara M. Antitumor activity of pembrolizumab in biomarker-unselected patients with recurrent and/or metastatic head and neck squamous cell carcinoma: results from the phase Ib KEYNOTE-012 expansion *Cohort*. (2016) 34:3838–45. 10.1200/JCO.2016.68.1478 27646946 PMC6804896

[13] Nivolumab doubles survival for patients with HNSCC Cancer Discov. (2016) 6:OF3 10.1158/2159-8290.CD-NB2016-049 27217382

[14] D'Angelo SP Russell J Lebbé C Chmielowski B Gambichler T Grob JJ. Efficacy and safety of first-line avelumab treatment in patients with stage IV metastatic merkel cell carcinoma: a preplanned interim analysis of a clinical trial. JAMA Oncol. (2018) 4:e180077. 10.1001/jamaoncol.2018.0077 29566106 PMC5885245

[15] Powles T O'Donnell PH Massard C Arkenau HT Friedlander TW Hoimes CJ Efficacy and safety of durvalumab in locally advanced or metastatic urothelial carcinoma: updated results from a phase 1/2 open-label study. JAMA Oncol. (2017) 3:e172411 10.1001/jamaoncol.2017.2411 28817753 PMC5824288

[16] Le DT Uram JN Wang H Bartlett BR Kemberling H Eyring AD. PD-1 blockade in tumors with mismatch-repair deficiency. N Engl J Med. (2015) 372:2509–20. 10.1056/NEJMoa1500596 26028255 PMC4481136

[17] Le DT Durham JN Smith KN Wang H Bartlett BR Aulakh LK. Mismatch repair deficiency predicts response of solid tumors to PD-1 blockade. Science. (2017) 357:409–13. 10.1126/science.aan6733 28596308 PMC5576142

[18] Overman MJ McDermott R Leach JL Lonardi S Lenz HJ Morse MA. Nivolumab in patients with metastatic DNA mismatch repair-deficient or microsatellite instability-high colorectal cancer (CheckMate 142): an open-label, multicentre, phase 2 study. Lancet Oncol. (2017) 18:1182–91. 10.1016/S1470-2045(17)30422-9 28734759 PMC6207072

[19] Herbst RS Soria JC Kowanetz M Fine GD Hamid O Gordon MS. Predictive correlates of response to the anti-PD-L1 antibody MPDL3280A in cancer patients. Nature. (2014) 515:563–7. 10.1038/nature14011 25428504 PMC4836193

[20] Gandini S Massi D Mandalà M. PD-L1 expression in cancer patients receiving anti PD-1/PD-L1 antibodies: a systematic review and meta-analysis. Crit Rev Oncol Hematol. (2016) 100:88–98. 10.1016/j.critrevonc.2016.02.001 26895815

[21] Aguiar PN Jr, De Mello RA, Hall P, Tadokoro H, Lima Lopes G. PD-L1 expression as a predictive biomarker in advanced non-small-cell lung cancer: updated survival data. Immunotherapy. (2017) 9:499–506. 10.2217/imt-2016-0150 28472902

[22] Chen L. Co-inhibitory molecules of the B7-CD28 family in the control of T-cell immunity. Nat Rev Immunol. (2004) 4:336–47. 10.1038/nri1349 15122199

[23] Townsend SE Allison JP. Tumor rejection after direct costimulation of CD8+ T cells by B7-transfected melanoma cells. Science. (1993) 259:368–70. 10.1126/science.7678351 7678351

[24] Walunas TL Lenschow DJ Bakker CY Linsley PS Freeman GJ Green JM. CTLA-4 can function as a negative regulator of T cell activation. Immunity. (1994) 1:405–13. 10.1016/1074-7613(94)90071-X 7882171

[25] Chen J Jiang CC Jin L Zhang XD. Regulation of PD-L1: a novel role of pro-survival signalling in cancer. Ann Oncol. (2016) 27:409–16. 10.1093/annonc/mdv615 26681673

[26] Chen L Han X. Anti-PD-1/PD-L1 therapy of human cancer: past, present, and future. J Clin Invest. (2015) 125:3384–91. 10.1172/JCI80011 26325035 PMC4588282

[27] Blank C Mackensen A. Contribution of the PD-L1/PD-1 pathway to T-cell exhaustion: an update on implications for chronic infections and tumor evasion. Cancer Immunol Immunother. (2007) 56:739–45. 10.1007/s00262-006-0272-1 17195077 PMC11030209

[28] Iwai Y Ishida M Tanaka Y Okazaki T Honjo T Minato N. Involvement of PD-L1 on tumor cells in the escape from host immune system and tumor immunotherapy by PD-L1 blockade. Proc Natl Acad Sci USA. (2002) 99:12293–7. 10.1073/pnas.192461099 12218188 PMC129438

[29] Keir ME Butte MJ Freeman GJ Sharpe AH. PD-1 and its ligands in tolerance and immunity. Annu Rev Immunol. (2008) 26:677–704. 10.1146/annurev.immunol.26.021607.090331 18173375 PMC10637733

[30] Zou W Chen L. Inhibitory B7-family molecules in the tumour microenvironment. Nat Rev Immunol. (2008) 8:467–77. 10.1038/nri2326 18500231

[31] Mazanet MM Hughes CC. B7-H1 is expressed by human endothelial cells and suppresses T cell cytokine synthesis. J Immunol. (2002) 169:3581–8. 10.4049/jimmunol.169.7.3581 12244148

[32] Ishida M Iwai Y Tanaka Y Okazaki T Freeman GJ Minato N. Differential expression of PD-L1 and PD-L2, ligands for an inhibitory receptor PD-1, in the cells of lymphohematopoietic tissues. Immunol Lett. (2002) 84:57–62. 10.1016/S0165-2478(02)00142-6 12161284

[33] Ingram JR Dougan M Rashidian M Knoll M Keliher EJ Garrett S. PD-L1 is an activation-independent marker of brown adipocytes. Nat Commun. (2017) 8:647. 10.1038/s41467-017-00799-8 28935898 PMC5608754

[34] Hino R Kabashima K Kato Y Yagi H Nakamura M Honjo T. Tumor cell expression of programmed cell death-1 ligand 1 is a prognostic factor for malignant melanoma. Cancer. (2010) 116:1757–66. 10.1002/cncr.24899 20143437

[35] Hamanishi J Mandai M Iwasaki M Okazaki T Tanaka Y Yamaguchi K. Programmed cell death 1 ligand 1 and tumor-infiltrating CD8+ T lymphocytes are prognostic factors of human ovarian cancer. Proc Natl Acad Sci USA. (2007) 104:3360–5. 10.1073/pnas.0611533104 17360651 PMC1805580

[36] Mu CY Huang JA Chen Y Chen C Zhang XG. High expression of PD-L1 in lung cancer may contribute to poor prognosis and tumor cells immune escape through suppressing tumor infiltrating dendritic cells maturation. Med Oncol. (2011) 28:682–8. 10.1007/s12032-010-9515-2 20373055

[37] Thompson RH Kuntz SM Leibovich BC Dong H Lohse CM Webster WS. Tumor B7-H1 is associated with poor prognosis in renal cell carcinoma patients with long-term follow-up. Cancer Res. (2006) 66:3381–5. 10.1158/0008-5472.CAN-05-4303 16585157

[38] Butte MJ Keir ME Phamduy TB Sharpe AH Freeman GJ. Programmed death-1 ligand 1 interacts specifically with the B7-1 costimulatory molecule to inhibit T cell responses. Immunity. (2007) 27:111–22. 10.1016/j.immuni.2007.05.016 17629517 PMC2707944

[39] Rollins MR Gibbons Johnson RM. CD80 expressed by CD8(+) T cells contributes to PD-L1-induced apoptosis of activated CD8(+) T cells. J Immunol Res. (2017) 2017:7659462. 10.1155/2017/7659462 29181416 PMC5664331

[40] Matsubara T Takada K Azuma K Takamori S Toyokawa G Haro A. A clinicopathological and prognostic analysis of PD-L2 expression in surgically resected primary lung squamous cell carcinoma. Ann Surg Oncol. (2019) 26:1925–33. 10.1245/s10434-019-07257-3 30815803

[41] Yearley JH Gibson C Yu N Moon C Murphy E Juco J. PD-L2 expression in human tumors: relevance to anti-PD-1 therapy in cancer. Clin Cancer Res. (2017) 23:3158–67. 10.1158/1078-0432.CCR-16-1761 28619999

[42] George S Papanicolau-Sengos A Lenzo FL Conroy JM Nesline M Pabla S. PD-L2 amplification and durable disease stabilization in patient with urothelial carcinoma receiving pembrolizumab. Oncoimmunology. (2018) 7:e1460298. 10.1080/2162402X.2018.1460298 30524881 PMC6279415

[43] Shibahara D Tanaka K Iwama E Kubo N Ota K Azuma K. Intrinsic and extrinsic regulation of PD-L2 expression in oncogene-driven non-small cell lung cancer. J Thorac Oncol. (2018) 13:926–37. 10.1016/j.jtho.2018.03.012 29596910

[44] Menguy S Prochazkova-Carlotti M Beylot-Barry M Saltel F Vergier B Merlio JP. PD-L1 and PD-L2 are differentially expressed by macrophages or tumor cells in primary cutaneous diffuse large B-cell lymphoma, leg type. Am J Surg Pathol. (2018) 42:326–34. 10.1097/PAS.0000000000000983 29112015

[45] Zhao SG Lehrer J Chang SL Das R Erho N Liu Y. The immune landscape of prostate cancer and nomination of PD-L2 as a potential therapeutic target. J Natl Cancer Inst. (2019) 111:301–10. 10.1093/jnci/djy141 30321406

[46] Takamori S Takada K Toyokawa G Azuma K Shimokawa M Jogo T. PD-L2 expression as a potential predictive biomarker for the response to anti-PD-1 drugs in patients with non-small cell lung cancer. Anticancer Res. (2018) 38:5897–901. 10.21873/anticanres.12933 30275216

[47] Budczies J Bockmayr M Denkert C Klauschen F Gröschel S Darb-Esfahani S. Pan-cancer analysis of copy number changes in programmed death-ligand 1 (PD-L1, CD274) - associations with gene expression, mutational load, and survival. Genes Chromosomes Cancer. (2016) 55:626–39. 10.1002/gcc.22365 27106868

[48] Roemer MG Advani RH Ligon AH Natkunam Y Redd RA Homer H. PD-L1 and PD-L2 genetic alterations define classical hodgkin lymphoma and predict outcome. J Clin Oncol. (2016) 34:2690–7. 10.1200/JCO.2016.66.4482 27069084 PMC5019753

[49] Green MR Monti S Rodig SJ Juszczynski P Currie T O'Donnell E. Integrative analysis reveals selective 9p24.1 amplification, increased PD-1 ligand expression, and further induction via JAK2 in nodular sclerosing Hodgkin lymphoma and primary mediastinal large B-cell lymphoma. Blood. (2010) 116:3268–77. 10.1182/blood-2010-05-282780 20628145 PMC2995356

[50] Budczies J Mechtersheimer G Denkert C Klauschen F Mughal SS Chudasama P. PD-L1 (CD274) copy number gain, expression, and immune cell infiltration as candidate predictors for response to immune checkpoint inhibitors in soft-tissue sarcoma. Oncoimmunology. (2017) 6:e1279777. 10.1080/2162402X.2017.1279777 28405504 PMC5384369

[51] Goldmann T Kugler C Reinmuth N Vollmer E Reck M. PD-L1 copy number gain in nonsmall-cell lung cancer defines a new subset of patients for anti PD-L1 therapy. Ann Oncol. (2016) 27:206–7. 10.1093/annonc/mdv510 26487587

[52] Ikeda S Okamoto T Okano S Umemoto Y Tagawa T Morodomi Y. PD-L1 is upregulated by simultaneous amplification of the PD-L1 and JAK2 genes in non-small cell lung cancer. J Thorac Oncol. (2016) 11:62–71. 10.1016/j.jtho.2015.09.010 26762740

[53] George J Saito M Tsuta K Iwakawa R Shiraishi K Scheel AH. Genomic amplification of CD274 (PD-L1) in small-cell lung cancer. Clin Cancer Res. (2017) 23:1220–6. 10.1158/1078-0432.CCR-16-1069 27620277 PMC6329376

[54] Georgiou K Chen L Berglund M Ren W de Miranda NF Lisboa S. Genetic basis of PD-L1 overexpression in diffuse large B-cell lymphomas. Blood. (2016) 127:3026–34. 10.1182/blood-2015-12-686550 27030389

[55] Wang W Sun J Li F Li R Gu Y Liu C. A frequent somatic mutation in CD274 3'-UTR leads to protein over-expression in gastric cancer by disrupting miR-570 binding. Hum Mutat. (2012) 33:480–4. 10.1002/humu.22014 22190470

[56] Wang W Li F Mao Y Zhou H Sun J Li R. A miR-570 binding site polymorphism in the B7-H1 gene is associated with the risk of gastric adenocarcinoma. Hum Genet. (2013) 132:641–8. 10.1007/s00439-013-1275-6 23430453

[57] Tao LH Zhou XR Li FC Chen Q Meng FY Mao Y. A polymorphism in the promoter region of PD-L1 serves as a binding-site for SP1 and is associated with PD-L1 overexpression and increased occurrence of gastric cancer. Cancer Immunol Immunother. (2017) 66:309–18. 10.1007/s00262-016-1936-0 27889799 PMC11028453

[58] Kataoka K Shiraishi Y Takeda Y Sakata S Matsumoto M Nagano S. Aberrant PD-L1 expression through 3'-UTR disruption in multiple cancers. Nature. (2016) 534:402–6. 10.1038/nature18294 27281199

[59] Kogure Y Kataoka K. Genetic alterations in adult T-cell leukemia/lymphoma. Cancer Sci. (2017) 108:1719–25. 10.1111/cas.13303 28627735 PMC5581529

[60] Wu Y Zhao T Jia Z Cao D Cao X Pan Y. Polymorphism of the programmed death-ligand 1 gene is associated with its protein expression and prognosis in gastric cancer. J Gastroenterol Hepatol. (2018). 10.1111/jgh.14520. [Epub ahead of print]. 30353572

[61] Esteller M. Epigenetics in cancer. N Engl J Med. (2008) 358:1148–59. 10.1056/NEJMra072067 18337604

[62] Jones PA Baylin SB. The fundamental role of epigenetic events in cancer. Nat Rev Genet. (2002) 3:415–28. 10.1038/nrg816 12042769

[63] Kim HJ Bae SC. Histone deacetylase inhibitors: molecular mechanisms of action and clinical trials as anti-cancer drugs. Am J Transl Res. (2011) 3:166–79. 21416059 PMC3056563

[64] Karpf AR Jones DA. Reactivating the expression of methylation silenced genes in human cancer. Oncogene. (2002) 21:5496–503. 10.1038/sj.onc.1205602 12154410

[65] Briere D Sudhakar N Woods DM Hallin J Engstrom LD Aranda R. The class I/IV HDAC inhibitor mocetinostat increases tumor antigen presentation, decreases immune suppressive cell types and augments checkpoint inhibitor therapy. Cancer Immunol Immunother. (2018) 67:381–92. 10.1007/s00262-017-2091-y 29124315 PMC11028326

[66] Héninger E Krueger TE Lang JM. Augmenting antitumor immune responses with epigenetic modifying agents. Front Immunol. (2015) 6:29. 10.3389/fimmu.2015.00029 25699047 PMC4316783

[67] Yang H Bueso-Ramos C DiNardo C Estecio MR Davanlou M Geng QR. Expression of PD-L1, PD-L2, PD-1 and CTLA4 in myelodysplastic syndromes is enhanced by treatment with hypomethylating agents. Leukemia. (2014) 28:1280–8. 10.1038/leu.2013.355 24270737 PMC4032802

[68] Kim TK Herbst RS Chen L. Defining and understanding adaptive resistance in cancer immunotherapy. Trends Immunol. (2018) 39:624–31. 10.1016/j.it.2018.05.001 29802087 PMC6066429

[69] Gevensleben H Holmes EE Goltz D Dietrich J Sailer V Ellinger J. PD-L1 promoter methylation is a prognostic biomarker for biochemical recurrence-free survival in prostate cancer patients following radical prostatectomy. Oncotarget. (2016) 7:79943–55. 10.18632/oncotarget.13161 27835597 PMC5346762

[70] Goltz D Gevensleben H Dietrich J Dietrich D. PD-L1 (CD274) promoter methylation predicts survival in colorectal cancer patients. Oncoimmunology. (2016) 6:e1257454. 10.1080/2162402X.2016.1257454 28197377 PMC5283627

[71] Goltz D Gevensleben H Grünen S Dietrich J Kristiansen G Landsberg J. PD-L1 (CD274) promoter methylation predicts survival in patients with acute myeloid leukemia. Leukemia. (2017) 31:738–43. 10.1038/leu.2016.328 27840427

[72] Micevic G Thakral D McGeary M Bosenberg M. PD-L1 methylation regulates PD-L1 expression and is associated with melanoma survival. Pigment Cell Melanoma Res. (2018) 32:435–40. 10.1111/pcmr.12745 30343532 PMC6475614

[73] Chatterjee A Rodger EJ Ahn A Stockwell PA Parry M Motwani J. Marked global DNA hypomethylation is associated with constitutive PD-L1 expression in melanoma. iScience. (2018) 4:312–25. 10.1016/j.isci.2018.05.021 30240750 PMC6147024

[74] Franzen A Vogt TJ Müller T Dietrich J Schröck A Golletz C. PD-L1 (CD274) and PD-L2 (PDCD1LG2) promoter methylation is associated with HPV infection and transcriptional repression in head and neck squamous cell carcinomas. Oncotarget. (2017) 9:641–50. 10.18632/oncotarget.23080 29416641 PMC5787495

[75] Berghoff AS Kiesel B Widhalm G Wilhelm D Rajky O Kurscheid S. Correlation of immune phenotype with IDH mutation in diffuse glioma. Neuro Oncol. (2017) 19:1460–8. 10.1093/neuonc/nox054 28531337 PMC5737620

[76] Mu L Long Y Yang C Jin L Tao H Ge H. The IDH1 mutation-induced oncometabolite. 2-hydroxyglutarate, may affect DNA methylation and expression of PD-L1 in gliomas. Front Mol Neurosci. (2018) 11:82. 10.3389/fnmol.2018.00082 29643764 PMC5882817

[77] Asgarova A Asgarov K Godet Y Peixoto P Nadaradjane A Boyer-Guittaut M. PD-L1 expression is regulated by both DNA methylation and NF-kB during EMT signaling in non-small cell lung carcinoma. Oncoimmunology. (2018) 7:e1423170. 10.1080/2162402X.2017.1423170 29721376 PMC5927541

[78] Wrangle J Wang W Koch A Easwaran H Mohammad HP Vendetti F. Alterations of immune response of non-small cell lung cancer with azacytidine. Oncotarget. (2013) 4:2067–79. 10.18632/oncotarget.1542 24162015 PMC3875770

[79] Lai Q Wang H Li A Xu Y Tang L Chen Q. Decitibine improve the efficiency of anti-PD-1 therapy via activating the response to IFN/PD-L1 signal of lung cancer cells. Oncogene. (2018) 37:2302–12. 10.1038/s41388-018-0125-3 29422611

[80] Zhang Y Xiang C Wang Y Duan Y Liu C Zhang Y. PD-L1 promoter methylation mediates the resistance response to anti-PD-1 therapy in NSCLC patients with EGFR-TKI resistance. Oncotarget. (2017) 8:101535–44. 10.18632/oncotarget.21328 29254184 PMC5731894

[81] Falkenberg KJ Johnstone RW. Histone deacetylases and their inhibitors in cancer, neurological diseases and immune disorders. Nat Rev Drug Discov. (2014) 13:673–91. 10.1038/nrd4360 25131830

[82] Zhan P Wang X Liu X Suzuki T. Medicinal chemistry insights into novel HDAC inhibitors: an updated patent review (2012-2016). Recent Pat Anticancer Drug Discov. (2017) 12:16–34. 10.2174/1574892811666161101102842 27804867

[83] Zheng H Zhao W Yan C Watson CC Massengill M Xie M. HDAC inhibitors enhance T-cell chemokine expression and augment response to PD-1 immunotherapy in lung adenocarcinoma. Clin Cancer Res. (2016) 22:4119–32. 10.1158/1078-0432.CCR-15-2584 26964571 PMC4987196

[84] Shen L Ciesielski M Ramakrishnan S Miles KM Ellis L Sotomayor P. Class I histone deacetylase inhibitor entinostat suppresses regulatory T cells and enhances immunotherapies in renal and prostate cancer models. PLoS ONE. (2012) 7:e30815. 10.1371/journal.pone.0030815 22303460 PMC3267747

[85] Kim K Skora AD Li Z Liu Q Tam AJ Blosser RL. Eradication of metastatic mouse cancers resistant to immune checkpoint blockade by suppression of myeloid-derived cells. Proc Natl Acad Sci USA. (2014) 111:11774–9. 10.1073/pnas.1410626111 25071169 PMC4136565

[86] Booth L Roberts JL Poklepovic A Kirkwood J Dent P. HDAC inhibitors enhance the immunotherapy response of melanoma cells. Oncotarget. (2017) 8:83155–70. 10.18632/oncotarget.17950 29137331 PMC5669957

[87] Wang YF Liu F Sherwin S Farrelly M Yan XG Croft A. Cooperativity of HOXA5 and STAT3 Is critical for HDAC8 inhibition-mediated transcriptional activation of PD-L1 in human melanoma cells. J Invest Dermatol. (2018) 138:922–32. 10.1016/j.jid.2017.11.009 29174371

[88] Woods DM Sodré AL Villagra A Sarnaik A Sotomayor EM Weber J. HDAC inhibition upregulates PD-1 ligands in melanoma and augments immunotherapy with PD-1 blockade. Cancer Immunol Res. (2015) 3:1375–85. 10.1158/2326-6066.CIR-15-0077-T 26297712 PMC4674300

[89] Lienlaf M Perez-Villarroel P Knox T Pabon M Sahakian E Powers J Essential role of HDAC6 in the regulation of PD-L1 in melanoma. Mol Oncol. (2016) 10:735–50. 10.1016/j.molonc.2015.12.012 26775640 PMC4870131

[90] Bae J Hideshima T Tai YT Song Y Richardson P Raje N. Histone deacetylase (HDAC) inhibitor ACY241 enhances anti-tumor activities of antigen-specific central memory cytotoxic T lymphocytes against multiple myeloma and solid tumors. Leukemia. (2018) 32:1932–47. 10.1038/s41375-018-0062-8 29487385 PMC6537609

[91] Booth L Roberts JL Poklepovic A Dent P. [pemetrexed + sildenafil], via autophagy-dependent HDAC downregulation, enhances the immunotherapy response of NSCLC cells. Cancer Biol Ther. (2017) 18:705–14. 10.1080/15384047.2017.1362511 28812434 PMC5663410

[92] Lu C Paschall AV Shi H Savage N Waller JL Sabbatini ME. The MLL1-H3K4me3 axis-mediated PD-L1 expression and pancreatic cancer immune evasion. J Natl Cancer Inst. (2017) 109:S587–89. 10.1093/jnci/djw283 28131992 PMC5291187

[93] Zhu H Bengsch F Svoronos N Rutkowski MR Bitler BG Allegrezza MJ. BET bromodomain inhibition promotes anti-tumor immunity by suppressing PD-L1 expression. Cell Rep. (2016) 16:2829–37. 10.1016/j.celrep.2016.08.032 27626654 PMC5177024

[94] Hogg SJ Vervoort SJ Deswal S Ott CJ Li J Cluse LA. BET-bromodomain inhibitors engage the host immune system and regulate expression of the immune checkpoint ligand PD-L1. Cell Rep. (2017) 18:2162–74. 10.1016/j.celrep.2017.02.011 28249162 PMC5340981

[95] Cioffi M Trabulo SM Vallespinos M Raj D Kheir TB Lin ML. The miR-25-93-106b cluster regulates tumor metastasis and immune evasion via modulation of CXCL12 and PD-L1. Oncotarget. (2017) 8:21609–25. 10.18632/oncotarget.15450 28423491 PMC5400610

[96] Bartel DP. MicroRNAs: target recognition and regulatory functions. Cell. (2009) 136:215–33. 10.1016/j.cell.2009.01.002 19167326 PMC3794896

[97] Tang D Zhao D Wu Y Yao R Zhou L Lu L The miR-3127-5p/p-STAT3 axis up-regulates PD-L1 inducing chemoresistance in non-small-cell lung cancer. J Cell Mol Med. (2018) 22:3847–56. 10.1111/jcmm.13657 PMC6050495 29726585

[98] Wang N Zhang T Down-regulation of microRNA-135 promotes sensitivity of non-small cell lung cancer to gefitinib by targeting TRIM16. Oncol Res. (2018) 26:1005–14. 10.3727/096504017X15144755633680 29295721 PMC7844745

[99] Zhu J Chen L Zou L Yang P Wu R Mao Y. MiR-20b,−21, and−130b inhibit PTEN expression resulting in B7-H1 over-expression in advanced colorectal cancer. Hum Immunol. (2014) 75:348–53. 10.1016/j.humimm.2014.01.006 24468585

[100] Wan J Ling X Peng B Ding G. miR-142-5p regulates CD4+ T cells in human non-small cell lung cancer through PD-L1 expression via the PTEN pathway. Oncol Rep. (2018) 40:272–82. 10.3892/or.2018.6439 29767245 PMC6059749

[101] Zhao L Yu H Yi S Peng X Su P Xiao Z. The tumor suppressor miR-138-5p targets PD-L1 in colorectal cancer. Oncotarget. (2016) 7:45370–84. 10.18632/oncotarget.9659 27248318 PMC5216728

[102] Xie WB Liang LH Wu KG Wang LX He X Song C. MiR-140 expression regulates cell proliferation and targets PD-L1 in NSCLC. Cell Physiol Biochem. (2018) 46:654–63. 10.1159/000488634 29617683

[103] Ji X Wang E Tian F. MicroRNA-140 suppresses osteosarcoma tumor growth by enhancing anti-tumor immune response and blocking mTOR signaling. Biochem Biophys Res Commun. (2018) 495:1342–8. 10.1016/j.bbrc.2017.11.120 29170130

[104] Dong P Xiong Y Yu J Chen L Tao T Yi S. Control of PD-L1 expression by miR-140/142/340/383 and oncogenic activation of the OCT4-miR-18a pathway in cervical cancer. Oncogene. (2018) 37:5257–68. 10.1038/s41388-018-0347-4 29855617 PMC6160397

[105] Naqvi AR Fordham JB Ganesh B Nares S. miR-24, miR-30b and miR-142-3p interfere with antigen processing and presentation by primary macrophages and dendritic cells. Sci Rep. (2016) 6:32925. 10.1038/srep32925 27611009 PMC5017188

[106] Jia L Xi Q Wang H Zhang Z Liu H Cheng Y. miR-142-5p regulates tumor cell PD-L1 expression and enhances anti-tumor immunity. Biochem Biophys Res Commun. (2017) 488:425–31. 10.1016/j.bbrc.2017.05.074 28511795

[107] Kao SC Cheng YY Williams M Kirschner MB Madore J Lum T. Tumor suppressor microRNAs contribute to the regulation of PD-L1 expression in malignant pleural mesothelioma. J Thorac Oncol. (2017) 12:1421–33. 10.1016/j.jtho.2017.05.024 28629895

[108] Yee D Shah KM Coles MC Sharp TV Lagos D. MicroRNA-155 induction via TNF-α and IFN-γ suppresses expression of programmed death ligand-1 (PD-L1) in human primary cells. J Biol Chem. (2017) 292:20683–93. 10.1074/jbc.M117.809053 29066622 PMC5733604

[109] Jia X Li X Shen Y Miao J Liu H Li G. MiR-16 regulates mouse peritoneal macrophage polarization and affects T-cell activation. J Cell Mol Med. (2016) 20:1898–907. 10.1111/jcmm.12882 27241533 PMC5020626

[110] Audrito V Serra S Stingi A Orso F Gaudino F Bologna C. PD-L1 up-regulation in melanoma increases disease aggressiveness and is mediated through miR-17-5p. Oncotarget. (2017) 8:15894–911. 10.18632/oncotarget.15213 28199980 PMC5362532

[111] He B Yan F Wu C. Overexpressed miR-195 attenuated immune escape of diffuse large B-cell lymphoma by targeting PD-L1. Biomed Pharmacother. (2018) 98:95–101. 10.1016/j.biopha.2017.11.146 29247952

[112] Ahn H Yang JM Kim H Chung JH Ahn SH Jeong WJ. Clinicopathologic implications of the miR-197/PD-L1 axis in oral squamous cell carcinoma. Oncotarget. (2017) 8:66178–94. 10.18632/oncotarget.19842 29029502 PMC5630402

[113] Fujita Y Yagishita S Hagiwara K Yoshioka Y Kosaka N Takeshita F. The clinical relevance of the miR-197/CKS1B/STAT3-mediated PD-L1 network in chemoresistant non-small-cell lung cancer. Mol Ther. (2015) 23:717–27. 10.1038/mt.2015.10 25597412 PMC4395779

[114] Sun C Lan P Han Q Huang M Zhang Z Xu G. Oncofetal gene SALL4 reactivation by hepatitis B virus counteracts miR-200c in PD-L1-induced T cell exhaustion. Nat Commun. (2018) 9:1241. 10.1038/s41467-018-03584-3 29593314 PMC5871883

[115] Pyzer AR Stroopinsky D Rosenblatt J Anastasiadou E Rajabi H Washington A. MUC1 inhibition leads to decrease in PD-L1levels via upregulation of miRNAs. Leukemia. (2017) 31:2780–90. 10.1038/leu.2017.163 28555079 PMC5791150

[116] Chen L Gibbons DL Goswami S Cortez MA Ahn YH Byers LA. Metastasis is regulated via microRNA-200/ZEB1 axis control of tumour cell PD-L1 expression and intratumoral immunosuppression. Nat Commun. (2014) 5:5241. 10.1038/ncomms6241 25348003 PMC4212319

[117] Xi J Huang Q Wang L Ma X Deng Q Kumar M. miR-21 depletion in macrophages promotes tumoricidal polarization and enhances PD-1 immunotherapy. Oncogene. (2018) 37:3151–65. 10.1038/s41388-018-0178-3 29540832 PMC5993583

[118] Miao S Mao X Zhao S Song K Xiang C Lv Y. miR-217 inhibits laryngeal cancer metastasis by repressing AEG-1 and PD-L1 expression. Oncotarget. (2017) 8:62143–53. 10.18632/oncotarget.19121 28977933 PMC5617493

[119] Boldrini L Giordano M Niccoli C Melfi F Lucchi M Mussi A. Role of microRNA-33a in regulating the expression of PD-1 in lung adenocarcinoma. Cancer Cell Int. (2017) 17:105. 10.1186/s12935-017-0474-y 29176936 PMC5693599

[120] Cortez MA Ivan C Valdecanas D Wang X Peltier HJ Ye Y. PDL1 Regulation by p53 via miR-34. J Natl Cancer Inst. (2015) 108:djv303. 10.1093/jnci/djv303 26577528 PMC4862407

[121] Wang Y Wang L. miR-34a attenuates glioma cells progression and chemoresistance via targeting PD-L1. Biotechnol Lett. (2017) 39:1485–92. 10.1007/s10529-017-2397-z 28721584

[122] Wang X Li J Dong K Lin F Long M Ouyang Y. Tumor suppressor miR-34a targets PD-L1 and functions as a potential immunotherapeutic target in acute myeloid leukemia. Cell Signal. (2015) 27:443–52. 10.1016/j.cellsig.2014.12.003 25499621

[123] Wu Q Zhao Y Sun Y Yan X Wang P. miR-375 inhibits IFN-γ-induced programmed death 1 ligand 1 surface expression in head and neck squamous cell carcinoma cells by blocking JAK2/STAT1 signaling. Oncol Rep. (2018) 39:1461–8. 10.3892/or.2018.6177 29328389

[124] Xu S Tao Z Hai B Liang H Shi Y Wang T. miR-424(322) reverses chemoresistance via T-cell immune response activation by blocking the PD-L1 immune checkpoint. Nat Commun. (2016) 7:11406. 10.1038/ncomms11406 27147225 PMC4858750

[125] Gong AY Zhou R Hu G Liu J Sosnowska D Drescher KM. Cryptosporidium parvum induces B7-H1 expression in cholangiocytes by down-regulating microRNA-513. J Infect Dis. (2010) 201:160–9. 10.1086/648589 19916867 PMC2791176

[126] Gong AY Zhou R Hu G Li X Splinter PL O'Hara SP. MicroRNA-513 regulates B7-H1 translation and is involved in IFN-γ-induced B7-H1 expression in cholangiocytes. J Immunol. (2009) 182:1325–33. 10.4049/jimmunol.182.3.1325 19155478 PMC2652126

[127] Wu L Chen Z Zhang J Xing Y. Effect of miR-513a-5p on etoposide-stimulating B7-H1 expression in retinoblastoma cells. J Huazhong Univ Sci Technolog Med Sci. (2012) 32:601–6. 10.1007/s11596-012-1004-8 22886978

[128] Zou MX Guo KM Lv GH Huang W Li J Wang XB. Clinicopathologic implications of CD8(+)/Foxp3(+) ratio and miR-574-3p/PD-L1 axis in spinal chordoma patients. Cancer Immunol Immunother. (2018) 67:209–24. 10.1007/s00262-017-2080-1 29051990 PMC11028121

[129] Halvorsen AR Sandhu V Sprauten M Flote VG Kure EH Brustugun OT. Circulating microRNAs associated with prolonged overall survival in lung cancer patients treated with nivolumab. Acta Oncol. (2018) 57:1225–31. 10.1080/0284186X.2018.1465585 29683761

[130] Zhang XL Xu LL Wang F. Hsa_circ_0020397 regulates colorectal cancer cell viability, apoptosis and invasion by promoting the expression of the miR-138 targets TERT and PD-L1. Cell Biol Int. (2017) 41:1056–64. 10.1002/cbin.10826 28707774

[131] Mastroianni J Stickel N Andrlová H Hanke K Melchinger W Duquesne S. miR-146a controls immune response in the melanoma microenvironment. Cancer Res. (2018) 79:183–95. 10.1158/0008-5472.CAN-18-1397 30425059 PMC6330089

[132] Okuma Y Hishima T Kashima J Homma S High PD-L1 expression indicates poor prognosis of HIV-infected patients with non-small cell lung cancer. Cancer Immunol Immunother. (2018) 67:495–505. 10.1007/s00262-017-2103-y 29243049 PMC11028207

[133] Said EA Al-Reesi I Al-Riyami M Al-Naamani K Al-Sinawi S Al-Balushi MS. Increased CD86 but Not CD80 and PD-L1 expression on liver CD68+ cells during chronic HBV Infection. PLoS ONE. (2016) 11:e0158265. 10.1371/journal.pone.0158265 27348308 PMC4922653

[134] Choschzick M Gut A Fink D. PD-L1 receptor expression in vulvar carcinomas is HPV-independent. Virchows Arch. (2018) 473:513–6. 10.1007/s00428-018-2364-7 29736798

[135] Outh-Gauer S Alt M Le Tourneau C Augustin J Broudin C Gasne C. Immunotherapy inhead and neck cancers: a new challenge for immunologists, pathologists and clinicians. Cancer Treat Rev. (2018) 65:54–64. 10.1016/j.ctrv.2018.02.008 29547766

[136] Severa M Giacomini E Gafa V Anastasiadou E Rizzo F Corazzari M. EBV stimulates TLR- and autophagy-dependent pathways and impairs maturation in plasmacytoid dendritic cells: implications forviral immune escape. Eur J Immunol. (2013) 43:147–58. 10.1002/eji.201242552 22996354

[137] Fang W Zhang J Hong S Zhan J Chen N Qin T. EBV-driven LMP1 and IFN-γ up-regulate PD-L1 in nasopharyngeal carcinoma: implications for oncotargeted therapy. Oncotarget. (2014) 5:12189–202. 10.18632/oncotarget.2608 25361008 PMC4322961

[138] Green MR Rodig S Juszczynski P Ouyang J Sinha P O'Donnell E. Constitutive AP-1 activity and EBV infection induce PD-L1 in Hodgkin lymphomas and posttransplant lymphoproliferative disorders: implications for targeted therapy. Clin Cancer Res. (2012) 18:1611–8. 10.1158/1078-0432.CCR-11-1942 22271878 PMC3321508

[139] Bass AJ Thorsson V Shmulevich I Reynolds SM Miller M Bernard B The cancer genome atlas research network. Comprehensive molecular characterization of gastric adenocarcinoma. Nature. (2014) 513:202–9. 10.1038/nature13480 25079317 PMC4170219

[140] Balsitis S Gali V Mason PJ Chaniewski S Levine SM Wichroski MJ. Safety and efficacy of anti-PD-L1 therapy in the woodchuck model of HBV infection. PLoS ONE. (2018) 13:e0190058. 10.1371/journal.pone.0190058 29444087 PMC5812555

[141] Park C Cho J Lee J Kang SY An JY Choi MG. Host immune response index in gastric cancer identified by comprehensive analyses of tumor immunity. Oncoimmunology. (2017) 6:e1356150. 10.1080/2162402X.2017.1356150 29147610 PMC5674954

[142] Abdellatif H Shiha G. PD-L1 expression on circulating CD34+ hematopoietic stem cells closely correlated with T-cell apoptosis in chronic hepatitis c infected patients. Int J Stem Cells. (2018) 11:78–86. 10.15283/ijsc17047 29291600 PMC5984061

[143] Ojiro K Qu X Cho H Park JJ Vuidepot A Lissin N. Modulation of hepatitis C virus-specific CD8 effector T-cell function with antiviral effect in infectious hepatitis C virus coculture model. J Virol. (2017) 91:e02129–16. 10.1128/JVI.02129-16 28275182 PMC5411593

[144] Choi YH Jin N Kelly F Sakthivel SK Yu T Elevation of alanine aminotransferase activity occurs after activation of the cell-death signaling initiated by pattern-recognition receptors ↱ but before activation of cytolytic effectors in NK or CD8^+^ T cells in the liver during acute HCV infection. PLoS ONE. (2016) 11:e0165533 10.1371/journal.pone.0165533 27788241 PMC5082795

[145] Fouad H Raziky MS Aziz RA Sabry D Aziz GM Ewais M. Dendritic cell co-stimulatory and co-inhibitory markers in chronic HCV: an Egyptian study. World J Gastroenterol. (2013) 19:7711–8. 10.3748/wjg.v19.i43.7711 24282359 PMC3837270

[146] Shen T Chen X Chen Y Xu Q Lu F Liu S. Increased PD-L1 expression and PD-L1/CD86 ratio on dendritic cells were associated with impaired dendritic cells function in HCV infection. J Med Virol. (2010) 82:1152–9. 10.1002/jmv.21809 20513078

[147] Domblides C Antoine M Hamard C Rabbe N Rodenas A Vieira T. Nonsmall cell lung cancer from HIV-infected patients expressed programmed cell death-ligand 1 with marked inflammatory infiltrates. AIDS. (2018) 32:461–8. 10.1097/QAD.0000000000001713 29194117

[148] Muthumani K Shedlock DJ Choo DK Fagone P Kawalekar OU Goodman J. HIV-mediated phosphatidylinositol 3-kinase/serine-threonine kinase activation in APCs leads to programmed death-1 ligand upregulation and suppression of HIV-specific CD8 T cells. J Immunol. (2011) 187:2932–43. 10.4049/jimmunol.1100594 21856939 PMC3197696

[149] Meier A Bagchi A Sidhu HK Alter G Suscovich TJ Kavanagh DG. Upregulation of PD-L1 on monocytes and dendritic cells by HIV-1 derived TLR ligands. AIDS. (2008) 22:655–8. 10.1097/QAD.0b013e3282f4de23 18317010 PMC2810187

[150] Planès R BenMohamed L Leghmari K Delobel P Izopet J Bahraoui E. HIV-1 Tatprotein induces PD-L1 (B7-H1) expression on dendritic cells through tumor necrosis factor alpha- and toll-like receptor 4-mediated mechanisms. J Virol. (2014) 88:6672–89. 10.1128/JVI.00825-14 24696476 PMC4054384

[151] Hong AM Vilain RE Romanes S Yang J Smith E Jones D. PD-L1 expression in tonsillar cancer is associated with human papillomavirus positivity and improved survival: implications for anti-PD1clinical trials. Oncotarget. (2016) 7:77010–20. 10.18632/oncotarget.12776 27776338 PMC5363566

[152] Lyford-Pike S Peng S Young GD Taube JM Westra WH Akpeng B. Evidence for a role of the PD-1: PD-L1 pathway in immune resistance of HPV-associated head and neck squamous cell carcinoma. Cancer Res. (2013) 73:1733–41. 10.1158/0008-5472.CAN-12-2384 23288508 PMC3602406

[153] Yang W Song Y Lu YL Sun JZ Wang HW. Increased expression of programmed death (PD)-1 and its ligand PD-L1 correlates with impaired cell-mediated immunity in high-risk human papillomavirus-related cervical intraepithelial neoplasia. Immunology. (2013) 139:513–22. 10.1111/imm.12101 23521696 PMC3719068

[154] Ukpo OC Thorstad WL Lewis JSJr B7-H1 expression model for immune evasion in human papilloma virus-related oropharyngeal squamous cell carcinoma. Head Neck Pathol. (2013) 7:113–21. 10.1007/s12105-012-0406-z 23179191 PMC3642256

[155] Lin PL Cheng YM Wu DW Huang YJ Lin HC Chen CY. A combination of anti-PD-L1 mAb plus Lm-LLO-E6 vaccine efficiently suppresses tumor growth and metastasis in HPV-infected cancers. Cancer Med. (2017) 6:2052–62. 10.1002/cam4.1143 28795532 PMC5603833

[156] Lipson EJ Vincent JG Loyo M Kagohara LT Luber BS Wang H. PD-L1 expression in the Merkel cell carcinoma microenvironment: association with inflammation, Merkel cell polyomavirus and overall survival. Cancer Immunol Res. (2013) 1:54–63. 10.1158/2326-6066.CIR-13-0034 24416729 PMC3885978

[157] Kebuchi R Konnai S Shirai T Sunden Y Murata S Onuma M Increase of cells expressing PD-L1 in bovine leukemia virus infection and enhancement of anti-viral immune responses in vitro via PD-L1 blockade. Vet Res. (2011) 42:103 10.1186/1297-9716-42-103 21943148 PMC3195098

[158] Host KM Jacobs SR West JA Zhang Z Costantini LM Stopford CM. Kaposi's sarcoma-associated herpesvirus increases PD-L1 and proinflammatory cytokine expression in human monocytes. MBio. (2017) 8:e00917-17. 10.1128/mBio.00917-17 29018115 PMC5635685

[159] Qian Y Deng J Geng L Xie H Jiang G Zhou L. TLR4 signaling induces B7-H1 expression through MAPK pathways in bladder cancer cells. Cancer Invest. (2008) 26:816–21. 10.1080/07357900801941852 18608206

[160] Huang B Zhao J Li H He KL Chen Y Chen SH. Toll-like receptors on tumor cells facilitate evasion of immune surveillance. Cancer Res. (2005) 65:5009–14. 10.1158/0008-5472.CAN-05-0784 15958541

[161] Beswick EJ Johnson JR Saada JI Humen M House J Dann S. TLR4 activation enhances the PD-L1-mediated tolerogenic capacity of colonic CD90+ stromal cells. J Immunol. (2014) 193:2218–29. 10.4049/jimmunol.1203441 25070848 PMC4142442

[162] Pulko V Liu X Krco CJ Harris KJ Frigola X Kwon ED. TLR3-stimulated dendritic cells up-regulate B7-H1 expression and influence the magnitude of CD8 T cell responses to tumor vaccination. J Immunol. (2009) 183:3634–41. 10.4049/jimmunol.0900974 19710456 PMC2789393

[163] Varthaman A Moreau HD Maurin M Benaroch P. TLR3-induced maturation of murine dendritic cells regulates CTL responses by modulating PD-L1 trafficking. PLoS ONE. (2016) 11:e0167057. 10.1371/journal.pone.0167057 27911948 PMC5135054

[164] Boes M Meyer-Wentrup F. TLR3 triggering regulates PD-L1 (CD274) expression in human neuroblastoma cells. Cancer Lett. (2015) 361:49–56. 10.1016/j.canlet.2015.02.027 25697485

[165] Gilardini Montani MS Santarelli R Falcinelli L Gonnella R Granato M DiRenzo L EBV up-regulates PD-L1 on the surface of primary monocytes by increasing ROS and activating TLR signaling and STAT3. J Leukoc Biol. (2018) 104:821–32. 10.1002/JLB.2A0118-029RR 30040158

[166] Bi XW Wang H Zhang WW Wang JH Liu WJ Xia ZJ. PD-L1 is upregulated by EBV-driven LMP1 through NF-κB pathway and correlates with poor prognosis in natural killer/T-cell lymphoma. J Hematol Oncol. (2016) 9:109. 10.1186/s13045-016-0341-7 27737703 PMC5064887

[167] Grinberg-Bleyer Y Ghosh S. A novel link between inflammation and cancer. Cancer Cell. (2016) 30:829–30. 10.1016/j.ccell.2016.11.013 27960080

[168] Wintterle S Schreiner B Mitsdoerffer M Schneider D Chen L Meyermann R. Expression of the B7-related molecule B7-H1 by glioma cells: a potential mechanism of immune paralysis. Cancer Res. (2003) 63:7462–7. 14612546

[169] Garcia-Diaz A Shin DS Moreno BH Saco J Escuin-Ordinas H Rodriguez GA. Interferon receptor signaling pathways regulating PD-L1 and PD-L2 expression. Cell Rep. (2017) 19:1189–201. 10.1016/j.celrep.2017.04.031 28494868 PMC6420824

[170] Kondo A Yamashita T Tamura H Zhao W Tsuji T Shimizu M Interferon-γ and tumor necrosis factor-alpha induce an immunoinhibitory molecule, B7-H1, via nuclear factor-kappa B activation in blasts in myelodysplastic syndromes. Blood. (2010) 116:1124–31. 10.1182/blood-2009-12-255125 20472834 PMC3375140

[171] Wang X Yang L Huang F Zhang Q Liu S Ma L. Inflammatory cytokines IL-17 and TNF-α up-regulate PD-L1 expression in human prostate and colon cancer cells. Immunol Lett. (2017) 184:7–14. 10.1016/j.imlet.2017.02.006 28223102 PMC5362328

[172] Quandt D Jasinski-Bergner S Müller U Schulze B Seliger B. Synergistic effects of IL-4 and TNFα on the induction of B7-H1 in renal cell carcinoma cells inhibiting allogeneic T cell proliferation. J Transl Med. (2014) 12:151. 10.1186/1479-5876-12-151 24885059 PMC4079621

[173] Li CW Lim SO Xia W Lee HH Chan LC Kuo CW. Glycosylation and stabilization of programmed death ligand-1 suppresses T-cell activity. Nat Commun. (2016) 7:12632. 10.1038/ncomms12632 27572267 PMC5013604

[174] Lastwika KJ Wilson W III Li QK Norris J Xu H Ghazarian SR. Control of PD-L1 expression by oncogenic activation of the AKT-mTOR pathway in non-small cell lung cancer. Cancer Res. (2016) 76:227–38. 10.1158/0008-5472.CAN-14-3362 26637667

[175] Carbotti G Barisione G Airoldi I Mezzanzanica D Bagnoli M Ferrero S. IL-27 induces the expression of IDO and PD-L1 in human cancer cells. Oncotarget. (2015) 6:43267–80. 10.18632/oncotarget.6530 26657115 PMC4791231

[176] Brown JA Dorfman DM Ma FR Sullivan EL Munoz O Wood CR. Blockade of programmed death-1 ligands on dendritic cells enhances T cell activation and cytokine production. J Immunol. (2003) 170:1257–66. 10.4049/jimmunol.170.3.1257 12538684

[177] de Kleijn S Langereis JD Leentjens J Kox M Netea MG Koenderman L IFN-γ-stimulated neutrophils suppress lymphocyte proliferation through expression of PD-L1. PLoS ONE. (2013) 8:e72249 10.1371/journal.pone.0072249 24015224 PMC3756078

[178] Schoop R Wahl P Le Hir M Heemann U Wang M Wüthrich RP. Suppressed T-cell activation by IFN-γ-induced expression of PD-L1 on renal tubular epithelial cells. Nephrol Dial Transplant. (2004) 19:2713–20. 10.1093/ndt/gfh423 15353579

[179] Noguchi T Ward JP Gubin MM Arthur CD Lee SH Hundal J. Temporally distinct PD-L1 expression by tumor and host cells contributes to immune escape. Cancer Immunol Res. (2017) 5:106–17. 10.1158/2326-6066.CIR-16-0391 28073774 PMC5510474

[180] Taube JM Anders RA Young GD Xu H Sharma R McMiller TL. Colocalization of inflammatory response with B7-h1 expression in human melanocytic lesions supports an adaptive resistance mechanism of immune escape. Sci Transl Med. (2012) 4:127ra37. 10.1126/scitranslmed.3003689 22461641 PMC3568523

[181] Abiko K Matsumura N Hamanishi J Horikawa N Murakami R Yamaguchi K. IFN-γ from lymphocytes induces PD-L1 expression and promotes progression of ovarian cancer. Br J Cancer. (2015) 112:1501–9. 10.1038/bjc.2015.101 25867264 PMC4453666

[182] Platanias LC. Mechanisms of type-I- and type-II-interferon-mediated signalling. Nat Rev Immunol. (2005) 5:375–86. 10.1038/nri1604 15864272

[183] Bellucci R Martin A Bommarito D Wang K Hansen SH Freeman GJ. Interferon-γ-induced activation of JAK1 and JAK2 suppresses tumor cell susceptibility to NK cells through upregulation of PD-L1 expression. Oncoimmunology. (2015) 4:e1008824. 10.1080/2162402X.2015.1008824 26155422 PMC4485824

[184] Gowrishankar K Gunatilake D Gallagher SJ Tiffen J Rizos H Hersey P Inducible but not constitutive expression of PD-L1 in human melanoma cells is dependent on activation of NF-κB. PLoS ONE. (2015) 10:e0123410 10.1371/journal.pone.0123410 25844720 PMC4386825

[185] Dorand RD Nthale J Myers JT Barkauskas DS Avril S Chirieleison SM. Cdk5 disruption attenuates tumor PD-L1 expression and promotes antitumor immunity. Science. (2016) 353:399–403. 10.1126/science.aae0477 27463676 PMC5051664

[186] Lim SO Li CW Xia W Cha JH Chan LC Wu Y. Deubiquitination and stabilization of PD-L1 by CSN5. Cancer Cell. (2016) 30:925–39. 10.1016/j.ccell.2016.10.010 27866850 PMC5171205

[187] Li N Wang J Zhang N Zhuang M Zong Z Zou J. Cross-talk between TNF-α and IFN-γ signaling in induction of B7-H1 expression in hepatocellular carcinoma cells. Cancer Immunol Immunother. (2018) 67:271–83. 10.1007/s00262-017-2086-8 29090321 PMC11028210

[188] Thomas DA Massagué J. TGF-β directly targets cytotoxic T cell functions during tumor evasion of immune surveillance. Cancer Cell. (2005) 8:369–80. 10.1016/j.ccr.2005.10.012 16286245

[189] Tauriello DVF Palomo-Ponce S Stork D Berenguer-Llergo A Badia-Ramentol J Iglesias M. TGFβ drives immune evasion in genetically reconstituted colon cancer metastasis. Nature. (2018) 554:538–43. 10.1038/nature25492 29443964

[190] Mariathasan S Turley SJ Nickles D Castiglioni A Yuen K Wang Y. TGFβ attenuates tumour response to PD-L1 blockade by contributing to exclusion of T cells. Nature. (2018) 554:544–8. 29443960 10.1038/nature25501 PMC6028240

[191] Kurimoto R Iwasawa S Ebata T Ishiwata T Sekine I Tada Y. Drug resistance originating from a TGF-β/FGF-2-driven epithelial-to-mesenchymal transition and its reversion in human lung adenocarcinoma cell lines harboring an EGFR mutation. Int J Oncol. (2016) 48:1825–36. 10.3892/ijo.2016.3419 26984042 PMC4809654

[192] Evanno E Godet J Piccirilli N Guilhot J Milin S Gombert JM. Tri-methylation of H3K79 is decreased in TGF-β1-induced epithelial-to-mesenchymal transition in lung cancer. Clin Epigenetics. (2017) 9:80. 10.1186/s13148-017-0380-0 28804523 PMC5549304

[193] Hsu JM Xia W Hsu YH Chan LC Yu WH Cha JH. STT3-dependent PD-L1 accumulation on cancer stem cells promotes immune evasion. Nat Commun. (2018) 9:1908. 10.1038/s41467-018-04313-6 29765039 PMC5954021

[194] Baas M Besançon A Goncalves T Valette F Yagita H Sawitzki B. TGFβ-dependent expression of PD-1 and PD-L1 controls CD8(+) T cell anergy in transplant tolerance. Elife. (2016) 5:e08133. 10.7554/eLife.08133 26824266 PMC4749558

[195] Lan Y Zhang D Xu C Hance KW Marelli B Qi J. Enhanced preclinical antitumor activity of M7824, a bifunctional fusionprotein simultaneously targeting PD-L1 and TGF-β. Sci Transl Med. (2018) 10:eaan5488. 10.1126/scitranslmed.aan5488 29343622

[196] Knudson KM Hicks KC Luo X Chen JQ Schlom J Gameiro SR. M7824, a novel bifunctional anti-PD-L1/TGFβ Trap fusion protein, promotes anti-tumor efficacy as monotherapy and in combination with vaccine. Oncoimmunology. (2018) 7:e1426519. 10.1080/2162402X.2018.1426519 29721396 PMC5927523

[197] Strauss J Heery CR Schlom J Madan RA Cao L Kang Z. Phase I trial of M7824 (MSB0011359C), a bifunctional fusion protein targeting PD-L1 and TGFβ, in advanced solid tumors. Clin Cancer Res. (2018) 24:1287–95. 10.1158/1078-0432.CCR-17-2653 29298798 PMC7985967

[198] Liu Y Ma Z Zhao C Wang Y Wu G Xiao J. HIF-1α and HIF-2α are critically involved in hypoxia-induced lipid accumulation in hepatocytes through reducing PGC-1α-mediated fatty acid β-oxidation. Toxicol Lett. (2014) 226:117–23. 10.1016/j.toxlet.2014.01.033 24503013

[199] Krzywinska E Stockmann C. Hypoxia, metabolism and immune cell function. Biomedicines. (2018) 6:56. 10.3390/biomedicines6020056 29762526 PMC6027519

[200] Barsoum IB Hamilton TK Li X Cotechini T Miles EA Siemens DR. Hypoxia induces escape from innate immunity in cancer cells via increased expression of ADAM10: role of nitric oxide. Cancer Res. (2011) 71:7433–41. 10.1158/0008-5472.CAN-11-2104 22006996

[201] Barsoum IB Smallwood CA Siemens DR Graham CH. A mechanism of hypoxia-mediated escape from adaptive immunity in cancer cells. Cancer Res. (2014) 74:665–74. 10.1158/0008-5472.CAN-13-0992 24336068

[202] Brown JM Wilson WR. Exploiting tumour hypoxia in cancer treatment. Nat Rev Cancer. (2004) 4:437–47. 10.1038/nrc1367 15170446

[203] Noman MZ Desantis G Janji B Hasmim M Karray S Dessen P. PD-L1 is a novel direct target of HIF-1α, and its blockade under hypoxia enhanced MDSC-mediated T cell activation. J Exp Med. (2014) 211:781–90. 10.1084/jem.20131916 24778419 PMC4010891

[204] Cubillos-Zapata C Avendaño-Ortiz J Hernandez-Jimenez E Toledano V Casas-Martin J Varela-Serrano A. Hypoxia-induced PD-L1/PD-1 crosstalk impairs T-cell function in sleep apnoea. Eur Respir J. (2017) 50:1700833. 10.1183/13993003.00833-2017 29051270

[205] Zhu Y Zang Y Zhao F Li Z Zhang J Fang L. Inhibition of HIF-1α by PX-478 suppresses tumor growth of esophageal squamous cell cancer *in vitro* and *in vivo*. Am J Cancer Res. (2017) 7:1198–212. 28560067 PMC5446484

[206] Avendaño-Ortiz J Maroun-Eid C Martín-Quirós A Toledano V Cubillos-Zapata C Gómez-Campelo P. PD-L1 overexpression during endotoxin tolerance impairs the adaptive immune response in septic patients via HIF1α. J Infect Dis. (2018) 217:393–404. 10.1093/infdis/jix279 28973671

[207] Koh J Jang JY Keam B Kim S Kim MY Go H. EML4-ALK enhances programmed cell death-ligand 1 expression in pulmonary adenocarcinoma via hypoxia-inducible factor (HIF)-1α and STAT3. Oncoimmunology. (2015) 5:e1108514. 10.1080/2162402X.2015.1108514 27141364 PMC4839370

[208] Ruf M Moch H Schraml P. PD-L1 expression is regulated by hypoxia inducible factor in clear cell renal cell carcinoma. Int J Cancer. (2016) 139:396–403. 10.1002/ijc.30077 26945902

[209] Messai Y Gad S Noman MZ Le Teuff G Couve S Janji B. Renal cell carcinoma programmed death-ligand 1, a new direct target of hypoxia-inducible factor-2 alpha, is regulated by von hippel-lindau gene mutation status. Eur Urol. (2016) 70:623–32. 10.1016/j.eururo.2015.11.029 26707870

[210] Huang Y Goel S Duda DG Fukumura D Jain RK. Vascular normalization as an emerging strategy to enhance cancer immunotherapy. Cancer Res. (2013) 73:2943–8. 10.1158/0008-5472.CAN-12-4354 23440426 PMC3655127

[211] Huang Y Yuan J Righi E Kamoun WS Ancukiewicz M Nezivar J. Vascular normalizing doses of antiangiogenic treatment reprogram the immunosuppressive tumor microenvironment and enhance immunotherapy. Proc Natl Acad Sci USA. (2012) 109:17561–6. 10.1073/pnas.1215397109 23045683 PMC3491458

[212] Koh YW Han JH Yoon DH Suh C Huh J. PD-L1 expression correlates with VEGF and microvessel density in patients with uniformly treated classical Hodgkin lymphoma. Ann Hematol. (2017) 96:1883–90. 10.1007/s00277-017-3115-6 28842748

[213] Meder L Schuldt P Thelen M Schmitt A Dietlein F Klein S. Combined VEGF and PD-L1 blockade displays synergistic treatment effects in an autochthonous mouse model of small cell lung cancer. Cancer Res. (2018) 78:4270–81. 10.1158/0008-5472.CAN-17-2176 29776963

[214] Socinski MA Jotte RM Cappuzzo F Orlandi F Stroyakovskiy D Nogami N. Atezolizumab for first-line treatment of metastatic nonsquamous NSCLC. N Engl J Med. (2018) 378:2288–301. 10.1056/NEJMoa1716948 29863955

[215] Choueiri TK Larkin J Oya M Thistlethwaite F Martignoni M Nathan P. Preliminary results for avelumab plus axitinib as first-line therapy in patients with advanced clear-cell renal-cell carcinoma (JAVELIN Renal 100): an open-label, dose-finding and dose-expansion, phase 1b trial. Lancet Oncol. (2018) 19:451–60. 10.1016/S1470-2045(18)30107-4 29530667

[216] McDermott DF Huseni MA Atkins MB Motzer RJ Rini BI Escudier B Clinical activity and molecular correlates of response to atezolizumab alone or in combination with bevacizumab versus sunitinib in renal cell carcinoma. Nat Med. (2018) 24:749–57. 10.1038/s41591-018-0053-3 29867230 PMC6721896

[217] Skinner HD Giri U Yang LP Kumar M Liu Y Story MD Integrative analysis identifies a novel AXL-PI3Kinase-PD-L1 signaling axis associated with radiation resistance in head and neck cancer. Clin Cancer Res. (2017) 23:2713–22. 10.1158/1078-0432.CCR-16-2586 28476872 PMC5457365

[218] Ishibashi M Tamura H Sunakawa M Kondo-Onodera A Okuyama N Hamada Y. Myeloma drug resistance induced by binding of myeloma B7-H1 (PD-L1) to PD-1. Cancer Immunol Res. (2016) 4:779–88. 10.1158/2326-6066.CIR-15-0296 27440711

[219] PD-L1 blockade maintains irradiation-mediated antitumor immunity Cancer Discov. (2014) 4:OF16 10.1158/2159-8290.CD-RW2014-012 24596210

[220] Zhang P Liu J Li W Li S Han X Lactoferricin B reverses cisplatin resistance in head and neck squamous cell carcinoma cells through targeting PD-L1. Cancer Med. (2018) 7:3178–87. 10.1002/cam4.1529 PMC6051176 29761938

[221] Jiang Z Yang Y Yang Y Zhang Y Yue Z Pan Z. Ginsenoside Rg3 attenuates cisplatin resistance in lung cancer by downregulating PD-L1 and resuming immune. Biomed Pharmacother. (2017) 96:378–83. 10.1016/j.biopha.2017.09.129 29031195

[222] Muraro E Furlan C Avanzo M Martorelli D Comaro E Rizzo A. Local high-dose radiotherapy induces systemic immunomodulating effects of potential therapeutic relevance in oligometastatic breast cancer. Front Immunol. (2017) 8:1476. 10.3389/fimmu.2017.01476 29163540 PMC5681493

[223] Dovedi SJ Adlard AL Lipowska-Bhalla G McKenna C Jones S Cheadle EJ. Acquired resistance to fractionated radiotherapy can be overcome by concurrent PD-L1 blockade. Cancer Res. (2014) 74:5458–68. 10.1158/0008-5472.CAN-14-1258 25274032

[224] Deng L Liang H Burnette B Beckett M Darga T Weichselbaum RR. Irradiation and anti-PD-L1 treatment synergistically promote antitumor immunityin mice. J Clin Invest. (2014) 124:687–95. 10.1172/JCI67313 24382348 PMC3904601

[225] D'Arrigo P Russo M Rea A Tufano M Guadagno E Del Basso De Caro ML. A regulatory role for the co-chaperone FKBP51s in PD-L1 expression in glioma. Oncotarget. (2017) 8:68291–304. 10.18632/oncotarget.19309 28978117 PMC5620257

[226] Song X Shao Y Jiang T Ding Y Xu B Zheng X. Radiotherapy upregulates programmed death Ligand-1 through the pathways downstream of epidermal growth factor receptor in glioma. EBio Medicine. (2018) 28:105–13. 10.1016/j.ebiom.2018.01.027 29396299 PMC5835577

[227] Kim HJ Park S Kim KJ Seong J. Clinical significance of soluble programmed cell death ligand-1 (sPD-L1) in hepatocellular carcinoma patients treated with radiotherapy. Radiother Oncol. (2018) 129:130–5. 10.1016/j.radonc.2017.11.027 29366520

[228] Oweida A Lennon S Calame D Korpela S Bhatia S Sharma J. Ionizing radiation sensitizes tumors to PD-L1 immune checkpoint blockade in orthotopic murine head and neck squamous cell carcinoma. Oncoimmunology. (2017) 6:e1356153. 10.1080/2162402X.2017.1356153 29123967 PMC5665079

[229] Sampath S Won H Massarelli E Li M Frankel P Vora N Combined modality radiation therapy promotes tolerogenic myeloidcell populations and STAT3-related gene expression in head and neck cancerpatients. Oncotarget. (2018) 9:11279–90. 10.18632/oncotarget.24397 29541413 PMC5834279

[230] Sato H Niimi A Yasuhara T Permata TBM Hagiwara Y Isono M. DNA double-strand break repair pathway regulates PD-L1 expression in cancer cells. Nat Commun. (2017) 8:1751. 10.1038/s41467-017-01883-9 29170499 PMC5701012

[231] Shen MJ Xu LJ Yang L Tsai Y Keng PC Chen Y. Radiation alters PD-L1/NKG2D ligand levels in lung cancer cells and leads to immune escape from NK cell cytotoxicity via IL-6-MEK/Erk signaling pathway. Oncotarget. (2017) 8:80506–20. 10.18632/oncotarget.19193 29113321 PMC5655216

[232] Azad A Yin Lim S D'Costa Z Jones K Diana A Sansom OJ. PD-L1 blockade enhances response of pancreatic ductal adenocarcinoma to radiotherapy. EMBO Mol Med. (2017) 9:167–80. 10.15252/emmm.201606674 27932443 PMC5286375

[233] Patel KR Martinez A Stahl JM Logan SJ Perricone AJ Ferris MJ. Increase in PD-L1 expression after pre-operative radiotherapy for soft tissue sarcoma. Oncoimmunology. (2018) 7:e1442168. 10.1080/2162402X.2018.1442168 29900051 PMC5993497

[234] Zhu B Tang L Chen S Yin C Peng S Li X. Targeting the upstream transcriptional activator of PD-L1 as an alternative strategy in melanoma therapy. Oncogene. (2018) 37:4941–54. 10.1038/s41388-018-0314-0 29786078

[235] Schutsky K Powell Jr. Daniel J (Perelman School of Medicine, University of Pennsylvania, Philadelphia, PA, USA). Unpublished observations: Chemotherapy and HDACi induce PD-L1 and Class I expression on ovarian cancer tumor cells. (2016).

[236] Rojkó L Reiniger L Téglási V Fábián K Pipek O Vágvölgyi A. Chemotherapy treatment is associated with altered PD-L1 expression in lung cancer patients. J Cancer Res Clin Oncol. (2018) 144:1219–26. 10.1007/s00432-018-2642-4 29675791 PMC11813485

[237] Qin X Liu C Zhou Y Wang G Cisplatin induces programmed death-1-ligand 1 (PD-L1) over-expression in hepatoma H22 cells via Erk /MAPK signaling pathway. Cell Mol Biol. (2010) 56(Suppl.)OL1366-72. 10.1170/156 20937224

[238] Tran L Allen CT Xiao R Moore E Davis R Park SJ. Cisplatin alters antitumor immunity and synergizes with PD-1/PD-L1 inhibition in head and neck squamous cell carcinoma. Cancer Immunol Res. (2017) 5:1141–51. 10.1158/2326-6066.CIR-17-0235 29097421 PMC5712281

[239] Ock CY Kim S Keam B Kim S Ahn YO Chung EJ. Changes in programmed death-ligand 1 expression during cisplatin treatment in patients with head and neck squamous cell carcinoma. Oncotarget. (2017) 8:97920–7. 10.18632/oncotarget.18542 29228662 PMC5716702

[240] Suda K Rozeboom L Rivard CJ Yu H Ellison K Melnick MAC. Therapy-induced E-cadherin downregulation alters expression of programmed death ligand-1 in lung cancer cells. Lung Cancer. (2017) 109:1–8. 10.1016/j.lungcan.2017.04.010 28577937 PMC6174882

[241] Shen M Tsai Y Zhu R Keng PC Chen Y Chen Y. FASN-TGF-β1-PD-L1 axis contributes to the development of resistance to NK cell cytotoxicity of cisplatin-resistant lung cancer cells. Biochim Biophys Acta. (2018) 1863:313–22. 10.1016/j.bbalip.2017.12.012 29306075

[242] Zhang P Ma Y Lv C Huang M Li M Dong B. Upregulation of programmed cell death ligand 1 promotes resistance response in non-small-cell lung cancer patients treated with neo-adjuvant chemotherapy. Cancer Sci. (2016) 107:1563–71. 10.1111/cas.13072 27581532 PMC5132280

[243] Yan F Pang J Peng Y Molina JR Yang P Liu S. Elevated cellular PD1/PD-L1 expression confers acquired resistance to cisplatin in small cell lung cancer cells. PLoS ONE. (2016) 11:e0162925. 10.1371/journal.pone.0162925 27610620 PMC5017656

[244] Yang M Liu P Wang K Glorieux C Hu Y Wen S. Chemotherapy induces tumor immune evasion by upregulation of programmed cell death ligand 1 expression in bone marrow stromal cells. Mol Oncol. (2017) 11:358–72. 10.1002/1878-0261.12032 28218497 PMC5527486

[245] Mesnage SJL Auguste A Genestie C Dunant A Pain E Drusch F. Neoadjuvant chemotherapy (NACT) increases immune infiltration and programmed death-ligand 1 (PD-L1) expression in epithelial ovarian cancer (EOC). Ann Oncol. (2017) 28:651–7. 10.1093/annonc/mdw625 27864219

[246] Ghebeh H Lehe C Barhoush E Al-Romaih K Tulbah A Al-Alwan M Doxorubicin downregulates cell surface B7-H1 expression and upregulates its nuclea rexpression in breast cancer cells: role of B7-H1 as an anti-apoptotic molecule. Breast Cancer Res. (2010) 12:R48 10.1186/bcr2605 20626886 PMC2949635

[247] Rom-Jurek EM Kirchhammer N Ugocsai P Ortmann O Wege AK Brockhoff G. Regulation of programmed death ligand 1 (PD-L1) expression in breast cancer cell lines *in vitro* and in immunodeficient and humanized tumor mice. Int J Mol Sci. (2018) 19:E563. 10.3390/ijms19020563 29438316 PMC5855785

[248] Zhang P Su DM Liang M Fu J. Chemopreventive agents induce programmed death-1-ligand 1 (PD-L1) surface expression in breast cancer cells and promote PD-L1-mediated T cell apoptosis. Mol Immunol. (2008) 45:1470–6. 10.1016/j.molimm.2007.08.013 17920123

[249] Doi T Ishikawa T Okayama T Oka K Mizushima K Yasuda T. The JAK/STAT pathway is involved in the upregulation of PD-L1 expression in pancreatic cancer cell lines. Oncol Rep. (2017) 37:1545–54. 10.3892/or.2017.5399 28112370

[250] Dosset M Vargas TR Lagrange A Boidot R Végran F Roussey A. PD-1/PD-L1 pathway: an adaptive immune resistance mechanism to immunogenic chemotherapy in colorectal cancer. Oncoimmunology. (2018) 7:e1433981. 10.1080/2162402X.2018.1433981 29872568 PMC5980491

[251] Gong W Song Q Lu X Gong W Zhao J Min P. Paclitaxel induced B7-H1 expression in cancer cells via the MAPK pathway. J Chemother. (2011) 23:295–9. 10.1179/joc.2011.23.5.295 22005063

[252] Peng J Hamanishi J Matsumura N Abiko K Murat K Baba T. Chemotherapy Induces Programmed Cell Death-Ligand 1 Overexpression via the Nuclear Factor-κB to Fosteran Immunosuppressive Tumor Microenvironment in Ovarian Cancer. Cancer Res. (2015) 75:5034–45. 10.1158/0008-5472.CAN-14-3098 26573793

[253] Guo Z Wang H Meng F Li J Zhang S. Combined trabectedin and anti-PD1antibody produces a synergistic antitumor effect in a murine model of ovarian cancer. J Transl Med. (2015) 13:247. 10.1186/s12967-015-0613-y 26219551 PMC4517526

[254] Van Der Kraak L Goel G Ramanan K Kaltenmeier C Zhang L Normolle DP 5-Fluorouracil upregulates cell surface B7-H1 (PD-L1) expression in gastrointestinal cancers. J Immunother Cancer. (2016) 4:65 10.1186/s40425-016-0163-8 27777774 PMC5067917

[255] Shalapour S Font-Burgada J Di Caro G Zhong Z Sanchez-Lopez E Dhar D. Immunosuppressive plasma cells impede T-cell-dependent immunogenic chemotherapy. Nature. (2015) 521:94–8. 10.1038/nature14395 25924065 PMC4501632

[256] Azuma K Ota K Kawahara A Hattori S Iwama E Harada T. Association of PD-L1 overexpression with activating EGFR mutations in surgically resected nonsmall-cell lung cancer. Ann Oncol. (2014) 25:1935–40. 10.1093/annonc/mdu242 25009014

[257] Jiang XM Xu YL Huang MY Zhang LL Su MX Chen X. Osimertinib (AZD9291) decreases programmed death ligand-1 in EGFR-mutated non-small cell lungcancer cells. Acta Pharmacol Sin. (2017) 38:1512–20. 10.1038/aps.2017.123 28880013 PMC5672073

[258] Liu L Mayes PA Eastman S Shi H Yadavilli S Zhang T. The BRAF and MEK inhibitors dabrafenib and trametinib: effects on immune function and in combination with immunomodulatory antibodies targeting PD-1, PD-L1, and CTLA-4. Clin Cancer Res. (2015) 21:1639–51. 10.1158/1078-0432.CCR-14-2339 25589619

[259] Jiang X Zhou J Giobbie-Hurder A Wargo J Hodi FS. The activation of MAPK in melanoma cells resistant to BRAF inhibition promotes PD-L1 expression that is reversible by MEK and PI3K inhibition. Clin Cancer Res. (2013) 19:598–609. 10.1158/1078-0432.CCR-12-2731 23095323

[260] Massi D Romano E Rulli E Merelli B Nassini R De Logu F. Baseline β-catenin, programmed death-ligand 1 expression and tumour-infiltrating lymphocytes predict response and poor prognosis in BRAF inhibitor-treated melanoma patients. Eur J Cancer. (2017) 78:70–81. 10.1016/j.ejca.2017.03.012 28412591

[261] Semaan A Dietrich D Bergheim D Dietrich J Kalff JC Branchi V. CXCL12 expression and PD-L1 expression serve as prognostic biomarkers in HCC and are induced by hypoxia. Virchows Arch. (2017) 470:185–96. 10.1007/s00428-016-2051-5 27913861

[262] Rice AE Latchman YE Balint JP Lee JH Gabitzsch ES Jones FR. An HPV-E6/E7 immunotherapy plus PD-1 checkpoint inhibition results in tumor regression and reduction in PD-L1 expression. Cancer Gene Ther. (2015) 22:454–62. 10.1038/cgt.2015.40 26337747

[263] Köhnke T Krupka C Tischer J Knösel T Subklewe M. Increase of PD-L1 expressing B-precursor ALL cells in a patient resistant to the CD19/CD3-bispecific T cell engager antibody blinatumomab. J Hematol Oncol. (2015) 8:111. 10.1186/s13045-015-0213-6 26449653 PMC4599591

[264] Yue C Jiang Y Li P Wang Y Xue J Li N. Dynamic change of PD-L1 expression on circulating tumor cells in advanced solid tumor patients undergoing PD-1 blockade therapy. Oncoimmunology. (2018) 7:e1438111. 10.1080/2162402X.2018.1438111 29900038 PMC5993493

[265] Dang CV. MYC on the path to cancer. Cell. (2012) 149:22–35. 10.1016/j.cell.2012.03.003 22464321 PMC3345192

[266] Casey SC Tong L Li Y Do R Walz S Fitzgerald KN. MYC regulates the antitumor immune response through CD47 and PD-L1. Science. (2016) 352:227–31. 10.1126/science.aac9935 26966191 PMC4940030

[267] Atsaves V Tsesmetzis N Chioureas D Kis L Leventaki V Drakos E. PD-L1 is commonly expressed and transcriptionally regulated by STAT3 and MYC in ALK-negative anaplastic large-cell lymphoma. Leukemia. (2017) 31:1633–7. 10.1038/leu.2017.103 28344319

[268] Wang J Jia Y Zhao S Zhang X Wang X Han X BIN1 reverses PD-L1-mediated immune escape by inactivating the c-MYC and EGFR/MAPK signaling pathways in non-small cell lung cancer. Oncogene. (2017) 6:6235–43. 10.1038/onc.2017.217 28714960

[269] Xu Y Poggio M Jin HY Shi Z Forester CM Wang Y. Translation control of the immune checkpoint in cancer and its therapeutic targeting. Nat Med. (2019) 25:301–11. 10.1038/s41591-018-0321-2 30643286 PMC6613562

[270] Shaulian E Karin M. AP-1 as a regulator of cell life and death. Nat Cell Biol. (2002) 4:E131–6. 10.1038/ncb0502-e131 11988758

[271] Sumimoto H Takano A Teramoto K Daigo Y RAS-mitogen-activated protein kinase signal is required for enhanced PD-L1 expression in human lung cancers. PLoS ONE. (2016) 1:e0166626 10.1371/journal.pone.0166626 PMC5112979 27846317

[272] Schaefer TS Sanders LK Nathans D Cooperative transcriptional activity of Jun and Stat3 beta, a short form of Stat3. Proc Natl Acad Sci USA. (1995) 2:9097–101. 10.1073/pnas.92.20.9097 PMC40931 7568080

[273] Zhang X Wrzeszczynska MH Horvath CM Darnell JE Jr Interacting regions in Stat3 and c-Jun that participate in cooperative transcriptional activation. MolCell Biol. (1999) 9:7138–46. 10.1128/MCB.19.10.7138 PMC84707 10490649

[274] Bu LL Yu GT Wu L Mao L Deng WW Liu JF STAT3 induces immunosuppression by upregulating PD-1/PD-L1 in HNSCC. J Dent Res. (2017) 6:1027–34. 10.1177/0022034517712435 PMC6728673 28605599

[275] Marzec M Zhang Q Goradia A Raghunath PN Liu X Paessler M Oncogenic kinase NPM/ALK induces through STAT3 expression of immunosuppressive protein CD274 (PD-L1, B7-H1). Proc Natl Acad Sci USA. (2008) 05:20852–7. 10.1073/pnas.0810958105 PMC2634900 19088198

[276] Concha-Benavente F Srivastava RM Trivedi S Lei Y Chandran U Seethala RR. Identification of the cell-intrinsic and –extrinsic pathways downstream of EGFR and IFNγ that induce PD-L1 expression in head and neck cancer. Cancer Res. (2016) 76:1031–43. 10.1158/0008-5472.CAN-15-2001 26676749 PMC4775348

[277] Pawlus MR Wang L Hu CJ STAT3 and HIF1α cooperatively activate HIF1 target genes in MDA-MB-231 and RCC4 cells. Oncogene. (2014) 3:1670–9. 10.1038/onc.2013.115 PMC3868635 23604114

[278] Bouillez A Rajabi H Jin C Samur M Tagde A Alam M MUC1-C integrates PD-L1 induction with repression of immune effectors in non-small-cell lung cancer. Oncogene. (2017) 6:4037–46. 10.1038/onc.2017.47 PMC5509481 28288138

[279] Roberts PJ Der CJ Targeting the Raf-MEK-ERK mitogen-activated protein kinase cascade for the treatment of cancer. Oncogene. (2007) 6:3291–310. 10.1038/sj.onc.1210422 17496923

[280] Coelho MA de Carné Trécesson S Rana S Zecchin D Moore C Molina-Arcas M Oncogenic RAS signaling promotes tumor immunoresistance by stabilizing PD-L1 mRNA. Immunity. (2017) 7:1083–99. 10.1016/j.immuni.2017.11.016 PMC5746170 29246442

[281] Chen N Fang W Lin Z Peng P Wang J Zhan J KRAS mutation-induced upregulation of PD-L1 mediates immune escape in human lung adenocarcinoma. Cancer Immunol Immunother. (2017) 6:1175–87. 10.1007/s00262-017-2005-z PMC5579171 28451792

[282] Atefi M Avramis E Lassen A Wong DJ Robert L Foulad D. Effects of MAPK and PI3K pathways on PD-L1 expression in melanoma. Clin Cancer Res. (2014) 20:3446–57. 10.1158/1078-0432.CCR-13-2797 24812408 PMC4079734

[283] Loi S Dushyanthen S Beavis PA Salgado R Denkert C Savas P RAS/MAPK activation is associated with reduced tumor-infiltrating lymphocytes in triple-negative breast cancer: therapeutic cooperation between MEK and PD-1/PD-L1 immune checkpoint inhibitors. Clin Cancer Res. (2016) 2:1499–509. 10.1158/1078-0432.CCR-15-1125 PMC4794351 26515496

[284] Minchom A Thavasu P Ahmad Z Stewart A Georgiou A O'Brien MER A study of PD-L1 expression in KRAS mutant non-small cell lung cancer cell lines exposed to relevant targeted treatments. PLoS ONE. (2017) 2:e0186106 10.1371/journal.pone.0186106 PMC5628934 28982179

[285] Chen N Fang W Zhan J Hong S Tang Y Kang S. Upregulation of PD-L1 by EGFR activation mediates the immune escape in EGFR-driven NSCLC: implication for optional immune targeted therapy for NSCLC patients with EGFR mutation. J Thorac Oncol. (2015) 10:910–23. 10.1097/JTO.0000000000000500 25658629

[286] Lin K Cheng J Yang T Li Y Zhu B EGFR-TKI down-regulates PD-L1 in EGFR mutant NSCLC through inhibiting NF-κB. Biochem Biophys Res Commun. (2015) 63:95–101. 10.1016/j.bbrc.2015.05.030 25998384

[287] Zhang N Zeng Y Du W Zhu J Shen D Liu Z The EGFR pathway is involved in the regulation of PD-L1 expression via the IL-6/JAK/STAT3 signaling pathway in EGFR-mutated non-small cell lung cancer. Int J Oncol. (2016) 9:1360–8. 10.3892/ijo.2016.3632 27499357

[288] Petrelli F Maltese M Tomasello G Conti B Borgonovo K Cabiddu M. Clinical and molecular predictors of PD-L1 expression in non-small-cell lung cancer: systematic review and meta-analysis. Clin Lung Cancer. (2018) 19:315–22. 10.1016/j.cllc.2018.02.006 29530732

[289] Roussel H De Guillebon E Biard L Mandavit M Gibault L Fabre E. Composite biomarkers defined by multiparametric immunofluorescence analysis identify ALK-positive adenocarcinoma as a potential target for immunotherapy. Oncoimmunology. (2017) 6:e1286437. 10.1080/2162402X.2017.1286437 28507793 PMC5414864

[290] Rangachari D VanderLaan PA Shea M Le X Huberman MS Kobayashi SS. Correlation between classic driver oncogene mutations in EGFR, ALK, or ROS1 and 22C3-PD-L1 ≥50% expression in lung adenocarcinoma. J Thorac Oncol. (2017) 12:878–3. 10.1016/j.jtho.2016.12.026 28104537 PMC5403565

[291] Toki MI Mani N Smithy JW Liu Y Altan M Wasserman B. Immune marker profiling and programmed death ligand 1 expression across NSCLC mutations. J Thorac Oncol. (2018) 13:1884–96. 10.1016/j.jtho.2018.09.012 30267840 PMC6251746

[292] Dong ZY Zhang JT Liu SY Su J Zhang C Xie Z. EGFR mutation correlates with uninflamed phenotype and weak immunogenicity, causing impaired response to PD-1 blockade in non-small cell lung cancer. Oncoimmunology. (2017) 6:e1356145. 10.1080/2162402X.2017.1356145 29147605 PMC5674946

[293] Song P Wu S Zhang L Zeng X Wang J. Correlation between PD-L1 expression and clinicopathologic features in 404 patients with lung adenocarcinoma. Interdiscip Sci. (2019). 10.1007/s12539-019-00329-8 31079342

[294] Hastings K Yu H Wei W Sanchez-Vega F DeVeaux M Choi J. EGFR mutation subtypes and response to immune checkpoint blockade treatment in non-small cell lung cancer. Ann Oncol. (2019). 10.1093/annonc/mdz141. [Epub ahead of print]. 31086949 PMC6683857

[295] Vivanco I Sawyers CL. The phosphatidylinositol 3-Kinase AKT pathway in human cancer. Nat Rev Cancer. (2002) 2:489–501. 10.1038/nrc839 12094235

[296] Parsa AT Waldron JS Panner A Crane CA Parney IF Barry JJ Loss of tumor suppressor PTEN function increases B7-H1 expression and immunoresistance in glioma. Nat Med. (2007) 3:84–8. 10.1038/nm1517 17159987

[297] Song M Chen D Lu B Wang C Zhang J Huang L. PTEN loss increases PD-L1 protein expression and affects the correlation between PD-L1 expression and clinical parameters in colorectal cancer. PLoS ONE. (2013) 8:e65821. 10.1371/journal.pone.0065821 23785454 PMC3681867

[298] McGowan M Hoven AS Lund-Iversen M Solberg S Helland Å Hirsch FR. PIK3CA mutations as prognostic factor in squamous cell lung carcinoma. Lung Cancer. (2017) 103:52–7. 10.1016/j.lungcan.2016.11.018 28024696

[299] Cerezo M Guemiri R Druillennec S Girault I Malka-Mahieu H Shen S. Translational control of tumor immune escape via the eIF4F-STAT1-PD-L1 axis in melanoma. Nat Med. (2018). 24:1877–86. 10.1038/s41591-018-0217-1 30374200

[300] Yamamoto R Nishikori M Tashima M Sakai T Ichinohe T Takaori-Kondo A. B7-H1 expression is regulated by MEK/ERK signaling pathway in anaplastic large cell lymphoma and Hodgkin lymphoma. Cancer Sci. (2009) 100:2093–100. 10.1111/j.1349-7006.2009.01302.x 19703193 PMC11158527

[301] Ota K Azuma K Kawahara A Hattori S Iwama E Tanizaki J. Induction of PD-L1 expression by the EML4-ALK oncoprotein and downstream signaling pathways in non-small cell lung cancer. Clin Cancer Res. (2015) 21:4014–21. 10.1158/1078-0432.CCR-15-0016 26019170

[302] Zanconato F Cordenonsi M Piccolo S YAP/TAZ at the roots of cancer. Cancer Cell. (2016) 9:783–803. 10.1016/j.ccell.2016.05.005 PMC6186419 27300434

[303] Moroishi T Hansen CG Guan KL The emerging roles of YAP and TAZ in cancer. Nat Rev Cancer. (2015) 5:73–9. 10.1038/nrc3876 PMC4562315 25592648

[304] Hsu PC Miao J Wang YC Zhang WQ Yang YL Wang CW Inhibition of yes-associated protein down-regulates PD-L1 (CD274) expression in human malignant pleural mesothelioma. J Cell Mol Med. (2018) 2:3139–48. 10.1111/jcmm.13593 PMC5980156 29575535

[305] Kim M. H. Kim C. G. Kim S. K. Shin S. J. Choe E. A. Park S. H. YAP-induced PD-L1 expression drives immune evasion in BRAFi-resistant melanoma. Cancer Immunol Res. (2018) 6:255–66. 10.1158/2326-6066.CIR-17-0320 29382670

[306] Janse van Rensburg HJ Azad T Ling M Hao Y Snetsinger B Khanal P The hippo pathway component TAZ promotes immune evasion in human cancer through PD-L1. Cancer Res. (2018) 8:1457–70. 10.1158/0008-5472.CAN-17-3139 29339539

[307] Miao J Hsu PC Yang YL Xu Z Dai Y Wang Y. YAP regulates PD-L1 expression in human NSCLC cells. Oncotarget. (2017) 8:114576–87. 10.18632/oncotarget.23051 29383103 PMC5777715

[308] Lee BS Park DI Lee DH Lee JE Yeo MK Park YH Hippo effector YAP directly regulates the expression of PD-L1 transcripts in EGFR-TKI-resistant lung adenocarcinoma. Biochem Biophys Res Commun. (2017) 91:493–9. 10.1016/j.bbrc.2017.07.007 28684311

[309] Feng J Yang H Zhang Y Wei H Zhu Z Zhu B Tumor cell-derived lactate induces TAZ-dependent upregulation of PD-L1 through GPR81 in human lung cancer cells. Oncogene. (2017) 6:5829–39. 10.1038/onc.2017.188 28604752

[310] Pfeifer GP Holmquist GP. Mutagenesis in the P53 gene. Biochim Biophys Acta. (1997) 1333:M1–8. 10.1016/S0304-419X(97)00004-8 9294014

[311] Canman CE Chen CY Lee MH Kastan MB. DNA damage responses: p53 induction, cell cycle perturbations, and apoptosis. Cold Spring Harb Symp Quant Biol. (1994) 59:277–86. 10.1101/SQB.1994.059.01.032 7587079

[312] Bensaad K Vousden KH. p53: new roles in metabolism. Trends Cell Biol. (2007) 17:286–91. 10.1016/j.tcb.2007.04.004 17481900

[313] Braun MW Iwakuma T. Regulation of cytotoxic T-cell responses by p53 in cancer. Transl Cancer Res. (2016) 5:692–7. 10.21037/tcr.2016.11.76 28944167 PMC5607642

[314] Muñoz-Fontela C Mandinova A Aaronson SA Lee SW. Emerging roles of p53 and other tumour-suppressor genes in immune regulation. Nat Rev Immunol. (2016) 16:741–50. 10.1038/nri.2016.99 27667712 PMC5325695

[315] Kim T Veronese A Pichiorri F Lee TJ Jeon YJ Volinia S. p53 regulates epithelial-mesenchymal transition through microRNAs targeting ZEB1 and ZEB2. J Exp Med. (2011) 208:875–83. 10.1084/jem.20110235 21518799 PMC3092351

[316] Kan G Dong W. The expression of PD-L1 APE1 and P53 in hepatocellular carcinoma and its relationship to clinical pathology. Eur Rev Med Pharmacol Sci. (2015) 19:3063–71. 26367730

[317] Dong ZY Zhong WZ Zhang XC Su J Xie Z Liu SY. Potential predictive value of TP53 and KRAS mutation status for response to PD-1 blockade immunotherapy in lung adenocarcinoma. Clin Cancer Res. (2017) 23:3012–24. 10.1016/j.jtho.2016.11.504 28039262

[318] Cha YJ Kim HR Lee CY Cho BC Shim HS. Clinicopathological and prognostic significance of programmed cell death ligand-1 expression in lung adenocarcinoma and its relationship with p53 status. Lung Cancer. (2016) 97:73–80. 10.1016/j.lungcan.2016.05.001 27237031

[319] Wieser V Gaugg I Fleischer M Shivalingaiah G Wenzel S Sprung S. BRCA1/2 and TP53 mutation status associates with PD-1 and PD-L1 expression in ovarian cancer. Oncotarget. (2018) 9:17501–11. 10.18632/oncotarget.24770 29707124 PMC5915132

[320] Yoon KW Byun S Kwon E Hwang SY Chu K Hiraki M. Control of signaling-mediated clearance of apoptotic cells by the tumor suppressor p53. Science. (2015) 349:1261669. 10.1126/science.1261669 26228159 PMC5215039

[321] Yu XY Zhang XW Wang F Lin YB Wang WD Chen YQ. Correlation and prognostic significance of PD-L1 and P53 expression in resected primary pulmonary lymphoepithelioma-like carcinoma. J Thorac Dis. (2018) 10:1891–902. 10.21037/jtd.2018.03.14 29707344 PMC5906333

[322] Li J Yen C Liaw D Podsypanina K Bose S Wang SI. PTEN, a putative protein tyrosine phosphatase gene mutated in human brain, breast, and prostate cancer. Science. (1997) 275:1943–7. 10.1126/science.275.5308.1943 9072974

[323] Myers MP Pass I Batty IH Van der Kaay J Stolarov JP Hemmings BA The lipid phosphatase activity of PTEN is critical forits tumor supressor function. Proc Natl Acad Sci USA. (1998) 95:13513–8. 10.1073/pnas.95.23.13513 9811831 PMC24850

[324] Mittendorf EA Philip AV Meric-Bernstam F Qiao N Wu Y Harrington S. PD-L1 expression in triple-negative breast cancer. Cancer Immunol Res. (2014) 2:361–70. 10.1158/2326-6066.CIR-13-0127 24764583 PMC4000553

[325] Wang X Cao X Sun R Tang C Tzankov A Zhang J. Clinical significance of PTEN deletion, mutation, and loss of PTEN expression in *de novo* diffuse large b-cell lymphoma. Neoplasia. (2018) 20:574–93. 10.1016/j.neo.2018.03.002 29734016 PMC5994742

[326] Buchakjian MR Merritt NM Moose DL Dupuy AJ Tanas MR Henry MD. A Trp53fl/fl Ptenfl/fl mouse model of undifferentiated pleomorphic sarcoma mediated by adeno-Cre injection and in vivo bioluminescence imaging. PLoS ONE. (2017) 12:e0183469. 10.1371/journal.pone.0183469 28841687 PMC5571905

[327] Xu C Fillmore CM Koyama S Wu H Zhao Y Chen Z. Loss of Lkb1 and Pten leads to lung squamous cell carcinoma with elevated PD-L1 expression. Cancer Cell. (2014) 25:590–604. 10.1016/j.ccr.2014.03.033 24794706 PMC4112370

[328] Best SA De Souza DP Kersbergen A Policheni AN Dayalan S Tull D. Synergy between the KEAP1/NRF2 and PI3K pathways drives non-small-cell lung cancer with an altered immune microenvironment. Cell Metab. (2018) 27:935–43. 10.1016/j.cmet.2018.02.006 29526543

[329] Ivanova AV Ivanov SV Prudkin L Nonaka D Liu Z Tsao A. Mechanisms of FUS1/TUSC2 deficiency in mesothelioma and its tumorigenic transcriptional effects. Mol Cancer. (2009) 8:91. 10.1186/1476-4598-8-91 19852844 PMC2776015

[330] Cao X Zhao Y Wang J Dai B Gentile E Lin J. TUSC2 downregulates PD-L1 expression in non-small cell lung cancer (NSCLC). Oncotarget. (2017) 8:107621–9. 10.18632/oncotarget.22581 29296193 PMC5746095

[331] Dai B Yan S Lara-Guerra H Kawashima H Sakai R Jayachandran G. Exogenous restoration of TUSC2 expression induces responsiveness to erlotinib in wildtype Epidermal Growth Factor Receptor (EGFR) lung cancer cells through context specific pathways resulting in enhanced therapeutic efficacy. PLoS ONE. (2015) 10:e0123967. 10.1371/journal.pone.0123967 26053020 PMC4460038

[332] Tan WJT Song L Graham M Schettino A Navaratnam D Yarbrough WG. Novel role of the mitochondrial protein fus1 in protection from premature hearing loss via regulation of oxidative stress and nutrient and energy sensing pathways in the inner ear. Antioxid Redox Signal. (2017) 27:489–509. 10.1089/ars.2016.6851 28135838 PMC5564041

[333] Manning AL Dyson NJ. RB: mitotic implications of a tumour suppressor. Nat Rev Cancer. (2012) 12:220–6. 10.1038/nrc3216 22318235 PMC3800194

[334] Schaal C Pillai S Chellappan SP. The Rb-E2F transcriptional regulatory pathway in tumor angiogenesis and metastasis. Adv Cancer Res. (2014) 121:147–82. 10.1016/B978-0-12-800249-0.00004-4 24889531

[335] Sherr CJ Roberts JM Living with or without cyclins and cyclin-dependent kinases. Genes Dev. (2004) 18:2699–711. 10.1101/gad.1256504 15545627

[336] Jin X Ding D Yan Y Li H Wang B Ma L. Phosphorylated RB promotes cancer immunity by inhibiting NF-κB activation and PD-L1 expression. Mol Cell. (2018) 73:22–35.e6. 10.1016/j.molcel.2018.10.034 30527665 PMC8968458

[337] Jeong JH Jo A Park P Lee H Lee HO. Brca2 deficiency leads to T cell loss and immune dysfunction. Mol Cells. (2015) 38:251–8. 10.14348/molcells.2015.2302 25666348 PMC4363725

[338] Strickland KC Howitt BE Shukla SA Rodig S Ritterhouse LL Liu JF. Association and prognostic significance of BRCA1/2-mutation status with neoantigen load, number of tumor-infiltrating lymphocytes and expression of PD-1/PD-L1 in high grade serous ovarian cancer. Oncotarget. (2016) 7:13587–98. 10.18632/oncotarget.7277 26871470 PMC4924663

[339] Dai Y Sun C Feng Y Jia Q Zhu B Potent immunogenicity in BRCA1-mutated patients with high-grade serous ovarian carcinoma. J Cell Mol Med. (2018) 22:3979–86. 10.1111/jcmm.13678 PMC6050488 29855141

[340] Horita H Law A Hong S Middleton K. Identifying regulatory posttranslational modifications of PD-L1: a focus on monoubiquitinaton. Neoplasia. (2017) 19:346–53. 10.1016/j.neo.2017.02.006 28319808 PMC5358937

[341] Cha JH Yang WH Xia W Wei Y Chan LC Lim SO. Metformin promotes antitumor immunity via endoplasmic-reticulum-associated degradation of PD-L1. Mol Cell. (2018) 71:606–620.e7. 10.1016/j.molcel.2018.07.030 30118680 PMC6786495

[342] Ding Q He X Hsu JM Xia W Chen CT Li LY Degradation of Mcl-1 by β-TrCP mediates glycogen synthase kinase3-induced tumor suppression and chemosensitization. Mol Cell Biol. (2007) 27:4006–17. 10.1128/MCB.00620-06 17387146 PMC1900029

[343] Hart M Concordet JP Lassot I Albert I del los Santos R Durand H. The F-box protein β-TrCP associates with phosphorylated β-catenin and regulates its activity in the cell. Curr Biol. (1999) 9:207–10. 10.1016/S0960-9822(99)80091-8 10074433

[344] Wang S Xu L Che X Li C Xu L Hou K. E3 ubiquitin ligases Cbl-b and c-Cbl downregulate PD-L1 in EGFR wild-type non-small cell lung cancer. FEBS Lett. (2018) 592:621–30. 10.1002/1873-3468.12985 29364514

[345] Zhang J Bu X Wang H Zhu Y Geng Y Nihira NT. Cyclin D-CDK4 kinase destabilizes PD-L1 via cullin 3-SPOP to control cancer immune surveillance. Nature. (2018) 553:91–5. 10.1038/nature25015 29160310 PMC5754234

[346] CDK4/6 inhibitors increase PD-L1 expression Cancer Discov. (2018) 8:12 10.1158/2159-8290.CD-RW2017-225

[347] Li J Yu T Yan M Zhang X Liao L Zhu M. DCUN1D1 facilitates tumor metastasis by activating FAK signaling and up-regulates PD-L1 in non-small-cell lung cancer. Exp Cell Res. (2018) 374:304–14. 10.1016/j.yexcr.2018.12.001 30528265

[348] Burr ML Sparbier CE Chan YC Williamson JC Woods K Beavis PA. CMTM6 maintains the expression of PD-L1 and regulates anti-tumour immunity. Nature. (2017) 549:101–5. 10.1038/nature23643 28813417 PMC5706633

[349] Mezzadra R Sun C Jae LT Gomez-Eerland R de Vries E Wu W. Identification of CMTM6 and CMTM4 as PD-L1 protein regulators. Nature. (2017) 549:106–10. 10.1038/nature23669 28813410 PMC6333292

[350] Visan I. CMTM6 controls PD-L1. Nat Immunol. (2017) 18:1067. 10.1038/ni.3844 28926544

[351] Wang H Yao H Li C Shi H Lan J Li Z. HIP1R targets PD-L1 to lysosomal degradation to alter T cell-mediated cytotoxicity. Nat Chem Biol. (2018) 15:42–50. 10.1038/s41589-018-0161-x 30397328

[352] Breitling J Aebi M. N-linked protein glycosylation in the endoplasmic reticulum. Cold Spring Harb Perspect Biol. (2013) 5:a013359. 10.1101/cshperspect.a013359 23751184 PMC3721281

[353] Schwarz F Aebi M. Mechanisms and principles of N-linked protein glycosylation. Curr Opin Struct Biol. (2011) 21:576–82. 10.1016/j.sbi.2011.08.005 21978957

[354] Li CW Lim SO Chung EM Kim YS Park AH Yao J. Eradication of triple-negative breast cancer cells by targeting glycosylated PD-L1. Cancer Cell. (2018) 33:187–201.e10. 10.1016/j.ccell.2018.01.009 29438695 PMC5824730

[355] Cheng X Veverka V Radhakrishnan A Waters LC Muskett FW Morgan SH. Structure and interactions of the human programmed cell death 1 receptor. J Biol Chem. (2013) 288:11771–85. 10.1074/jbc.M112.448126 23417675 PMC3636866

[356] Maher CM Thomas JD Haas DA Longen CG Oyer HM Tong JY. Small-molecule sigma1 modulator induces autophagic degradation of PD-L1. Mol Cancer Res. (2018) 16:243–55. 10.1158/1541-7786.MCR-17-0166 29117944

[357] Salatino M Girotti MR Rabinovich GA. Glycans pave the way for immunotherapy in triple-negative breast cancer. Cancer Cell. (2018) 33:155–7. 10.1016/j.ccell.2018.01.015 29438689

[358] Chen S Zhu B Yin C Liu W Han C Chen B. Palmitoylation-dependent activation of MC1R prevents melanomagenesis. Nature. (2017) 549:399–403. 10.1038/nature23887 28869973 PMC5902815

[359] Linder ME Deschenes RJ. Palmitoylation: policing protein stability and traffic. Nat Rev Mol Cell Biol. (2007) 8:74–84. 10.1038/nrm2084 17183362

[360] Li W Li W Zou L Ji S Li C Liu K. Membrane targeting of inhibitory Smads through palmitoylation controls TGF-β/BMP signaling. Proc Natl Acad Sci USA. (2017) 114:13206–11. 10.1073/pnas.1710540114 29180412 PMC5740658

[361] Blanc M David F Abrami L Migliozzi D Armand F Bürgi J. SwissPalm: protein palmitoylation database. F1000Res. (2015) 4:261. 10.12688/f1000research.6464.1 26339475 PMC4544385

[362] Yang Y Hsu JM Sun L Chan LC Li CW Hsu JL. Palmitoylation stabilizes PD-L1 to promote breast tumor growth. Cell Res. (2018) 29:83–6. 10.1038/s41422-018-0124-5 30514902 PMC6318320

[363] Schmidt MF Schlesinger MJ. Fatty acid binding to vesicular stomatitis virus glycoprotein: a new type of post-translational modification of the viral glycoprotein. Cell. (1979) 17:813–9. 10.1016/0092-8674(79)90321-0 226266

[364] Mitchell DA Vasudevan A Linder ME Deschenes RJ. Protein palmitoylation by a family of DHHC protein S-acyltransferases. J Lipid Res. (2006) 47:1118–27. 10.1194/jlr.R600007-JLR200 16582420

[365] Yao H Lan J Li C Shi H Brosseau JP Wang H Inhibiting PD-L1 palmitoylation enhances T-cell immune responses against tumours. Nat Biomed Eng. (2019) 3:306–17. 10.1038/s41551-019-0375-6 30952982

[366] Liu J Hamrouni A Wolowiec D Coiteux V Kuliczkowski K Hetuin D. Plasma cells from multiple myeloma patients express B7-H1 (PD-L1) and increase expression after stimulation with IFN-γ and TLR ligands via a MyD88-, TRAF6-, and MEK-dependent pathway. Blood. (2007) 110:296–304. 10.1182/blood-2006-10-051482 17363736

[367] Fei Z Deng Z Zhou L Li K Xia X Xie R. PD-L1 induces epithelial mesenchymal transition in nasopharyngeal carcinoma cells through activation PI3K/AKT pathway. Oncol Res. (2019). 10.3727/096504018X15446984186056. [Epub ahead of print]. 30982497 PMC7848446

[368] Tu X Qin B Zhang Y Zhang C Kahila M Nowsheen S PD-L1 (B7-H1) competes with the RNA exosome to regulate the DNA damage response and can be targeted to sensitize to radiation or chemotherapy. Mol Cell. (2019) 74:1–12. 10.1016/j.molcel.2019.04.005 31053471 PMC6737939

